# Flavonoids as modulators of gut-liver axis: emerging therapeutic strategies for MAFLD

**DOI:** 10.3389/fphar.2025.1657751

**Published:** 2025-10-16

**Authors:** Mengxuan Hao, Zihui Wang, Liren Wang, Aidiya Yimamu, Xiaoling Su, Minmin Zhang, Xincan Li, Quanlong Zhang, Zeyu Sun

**Affiliations:** ^1^ State Key Laboratory for Diagnosis and Treatment of Infectious Diseases, National Clinical Research Center for Infectious Diseases, China-Singapore Belt and Road Joint Laboratory on Infection Research and Drug Development, National Medical Center for Infectious Diseases, Collaborative Innovation Center for Diagnosis and Treatment of Infectious Diseases, The First Affiliated Hospital, Zhejiang University School of Medicine, Hangzhou, China; ^2^ Yuhang Institute of Medical Science Innovation and Transformation, Hangzhou, China; ^3^ Key Laboratory of Oral Biomedical Research of Zhejiang Province, Stomatology Hospital, School of Stomatology, Zhejiang University School of Medicine, Zhejiang Provincial Clinical Research Center for Oral Diseases, Cancer Center of Zhejiang University, Hangzhou, China; ^4^ College of Pharmaceutical Sciences, Zhejiang University, Hangzhou, China; ^5^ Jinan Microecological Biomedicine Shandong Laboratory, Jinan, China; ^6^ College of Pharmaceutical Sciences, Zhejiang Chinese Medical University, Hangzhou, China

**Keywords:** MAFLD, flavonoids, gut-liver axis, gut microbial modulation, natural herbal products

## Abstract

Metabolic dysfunction-associated fatty liver disease (MAFLD) is a significant global health challenge affecting approximately 25% of adults worldwide. Given the limited efficacy of existing therapies, there is an urgent need for novel treatment strategies. Flavonoids, a diverse class of natural polyphenolic compounds, exhibit significant potential in ameliorating MAFLD by modulating hepatic lipid metabolism and immune-inflammatory responses via gut-liver axis. This review systematically explores the interactions between flavonoids and gut microbiota, elucidating their role in MAFLD progression. We highlight how flavonoid structural diversity and microbial biotransformation modulate multiple key pathways, such as PPARα, PPARγ, ERβ, Nrf2, NF-κB, and FXR signalling. These multi-target mechanisms underpin the therapeutic potential of flavonoids in reducing lipid accumulation, oxidative stress, inflammation, and fibrosis in MAFLD. We also discuss innovative strategies, including flavonoid-probiotic synergies, nanotechnology-enhanced delivery systems, and personalized nutrition strategies. By integrating evidence from preclinical models and clinical trials, we highlight the translational potential of flavonoid-based interventions for MAFLD management. Our analysis underscores flavonoids as multi-target, safe and effective solutions for MAFLD management, warranting further clinical studies to translate these findings into routine clinical practice.

## 1 Introduction

Metabolic dysfunction-associated fatty liver disease (MAFLD) is a global health challenge affecting an estimated 25% of the adult, with its prevalence rising due to the increasing rates of obesity, diabetes, and metabolic syndrome (Younossi et al., 2023; Man et al., 2023). Its severe form, metabolic dysfunction-associated steatohepatitis (MASH), is a leading cause of liver cirrhosis and hepatocellular carcinoma (HCC) (Huang et al., 2021; Paik et al., 2022). Despite this significant burden, effective drug therapies for MAFLD are lacking, as many anticipated drugs have failed in clinical trials (Rong et al., 2023; Piero et al., 2024). Diet is a critical factor in MAFLD pathogenesis, with diets rich in refined sugars, unhealthy fats, and low in essential micronutrients exacerbating its progression (Francesca et al., 2022). Of particular interest is the role of flavonoid-rich diets, such as the Mediterranean diet, which has been consistently linked to a reduced risk of developing MAFLD (Ilaria et al., 2020; Riazi et al., 2022). Epidemiological studies have demonstrated that individuals with diets deficient in flavonoids are at an increased risk of developing MAFLD (Iino et al., 2022), further emphasizing the importance of dietary interventions in MAFLD management (Sherouk and Joseph, 2023).

**FIGURE 1:**
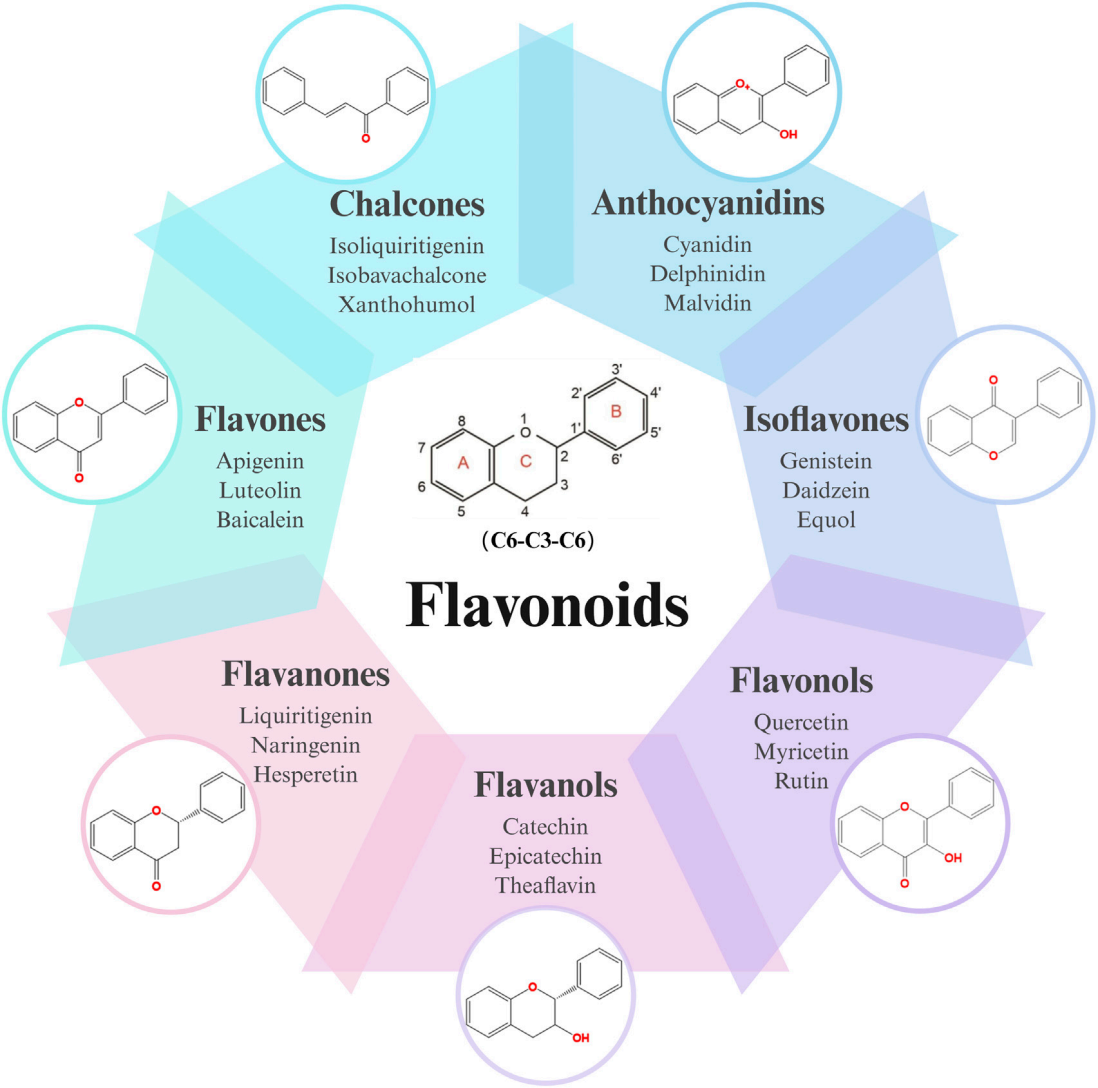
Skeletal structure of flavonoids and their seven subclassifications. Original image drawn for this review using Biorender software.

Flavonoids, a diverse group of natural polyphenolic compounds, have garnered attention as “food-derived medicine” therapeutic potential. These compounds exhibit remarkable structural diversity, characterized by their C6-C3-C6 backbone (Figure 1), which allows for a wide range of substitution patterns and bioactive properties (Dias et al., 2021; Kaushal et al., 2022). Flavonoids are abundant in citrus fruits, soy, tea, and medicinal herbals such as snow lotus (*Saussurea eriocephala* Franch), Chinese skullcap (*Scutellaria baicalensis* Georgi), *Dendrobium officinale* Kimura and Migo, Dragon’s blood (*Dracaena draco* (L.) L.) and Ficus hirta (*Ficus simplicissima* Lour) (Dias et al., 2021; Shen et al., 2022; Yi et al., 2012; Yi et al., 2009a; Chen et al., 2017; Xue et al., 2017; Yi et al., 2009b) (Table 1). Pharmacologically, flavonoids show potent antioxidant and anti-inflammatory properties by modulating oxidative stress pathways (e.g., Nrf2, CYP2E1, ROS) and inhibiting pro-inflammatory signalling pathways (e.g., NF-κB, TNF-α, TLR4) (Kaushal et al., 2022; Shen et al., 2022). As estrogen analogs, flavonoids may modulate diseases associated with estrogen abnormalities with low toxicity and low side effects (Kiyama, 2023). As food-derived medicine, flavonoids were found to modulate intestinal microbiota, which in turn affect host metabolism and immune function (Kuziel et al., 2025). After being ingested by the body, the metabolic fate of flavonoids is influenced by their structural characteristics and their interactions with the intestinal microbiota (Kuziel et al., 2025; Li C. et al., 2023). A major challenge lies in their typically low bioavailability and extensive first-pass metabolism, which severely limits the flavonoid concentration in systemic circulation and target tissues. Furthermore, the diverse metabolic transformations by gut microbiota and host enzymes can lead to varied, making their precise *in vivo* effects difficult to predict and standardize across individuals. Therefore, how to enhance the bioavailability of flavonoids within the context of gut-liver crosstalk is crucial for alleviating liver diseases such as MAFLD. In summary, flavonoids’ structural diversity, rich dietary sources, and broad spectrum of pharmacological activities position them as promising candidates for developing novel therapeutic strategies of MAFLD (Kaushal et al., 2022; Li C. et al., 2023; Xiaopeng et al., 2022).

**TABLE 1:** Structure, characteristics, food sources and representative molecules of the seven subclasses of flavonoids.

Flavonoids subclass	Structural features	Food source	MALFD related compounds	References
Flavones	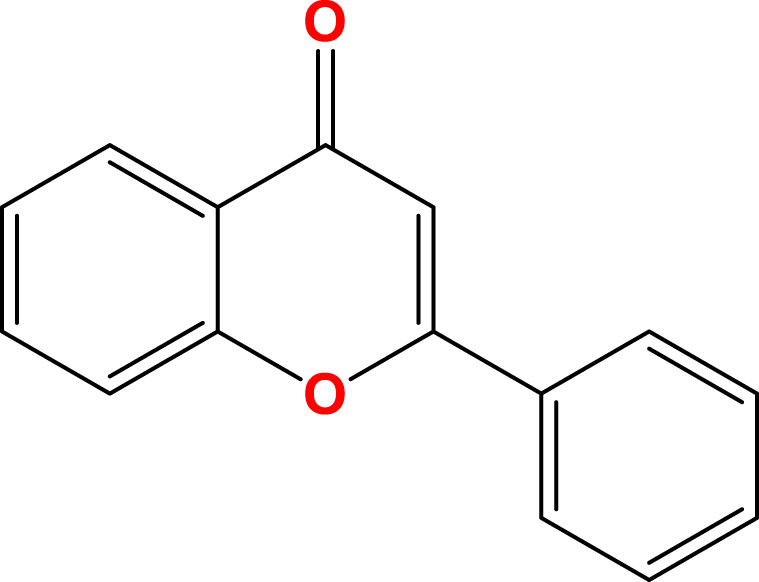 The chemical structure consists of 4H-chromen-4-one, which bears a phenyl substituent at position 2	Chamomile, Parsley, Lamiaceae, Bergamot, Tea leaves and Herbs	Apigenin, Luteolin, Baicalein	Jeong et al. (2022), Hostetler et al. (2017)
Flavanones (dihydroflavones)	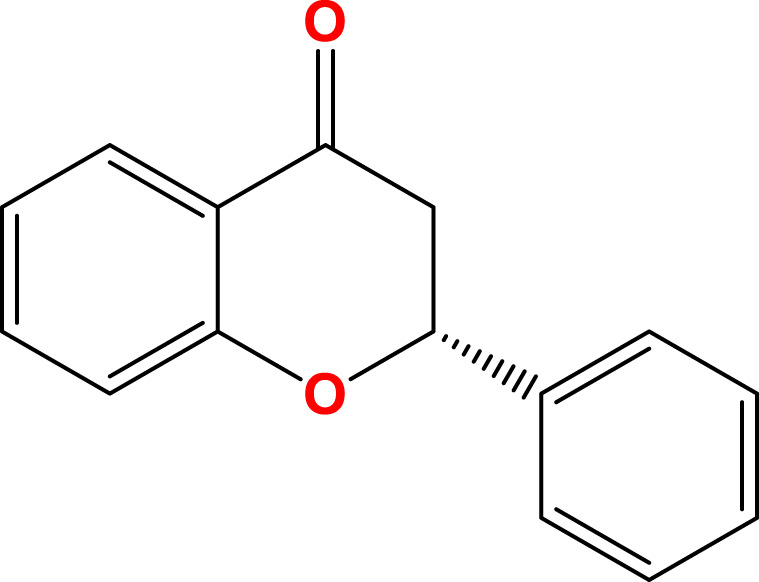 The C2 = C3 double bond	Citrus fruits (Grapefruits, Lemons, Oranges), Tomatoes, Cherries	Hesperetin, Naringenin, Liquiritigenin	Barreca et al. (2017), Motallebi et al. (2022)
Flavanols (flavan-3-ols)	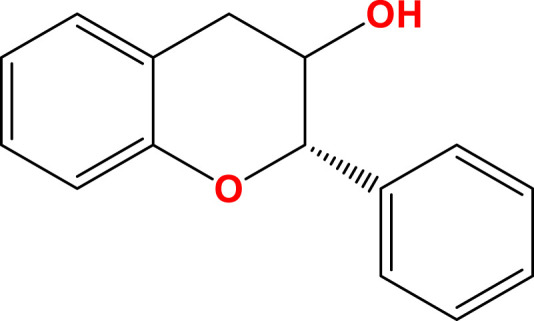 C-3 hydroxyl group	Cereals, Legumes, Forages, Hops, Beers, Red wine, Tea, Cocoa	(+)-Catechin, (−)-Epicatechin, Theaflavin	Luo et al. (2022), Martín and Ramos (2021), Ottaviani et al. (2018)
Flavonols (3-hydroxyflavone)	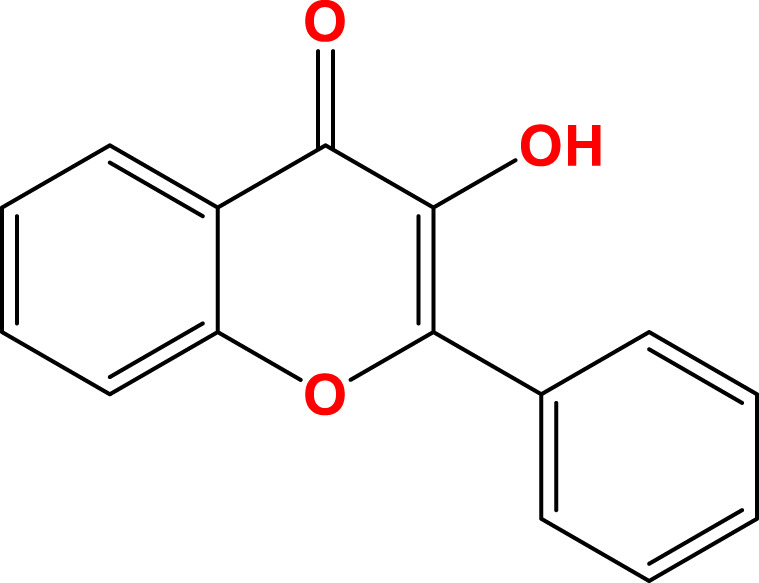 There are several specific substitutions in the A and B rings, which are connected by a three-carbon chain	Onions, Apples, Green tea, Berries, Nuts	Quercetin, Myricetin, Rutin	Jazvinšćak et al. (2023), Popiolek-Kalisz and Fornal (2022), Wendlocha et al. (2024)
Isoflavones	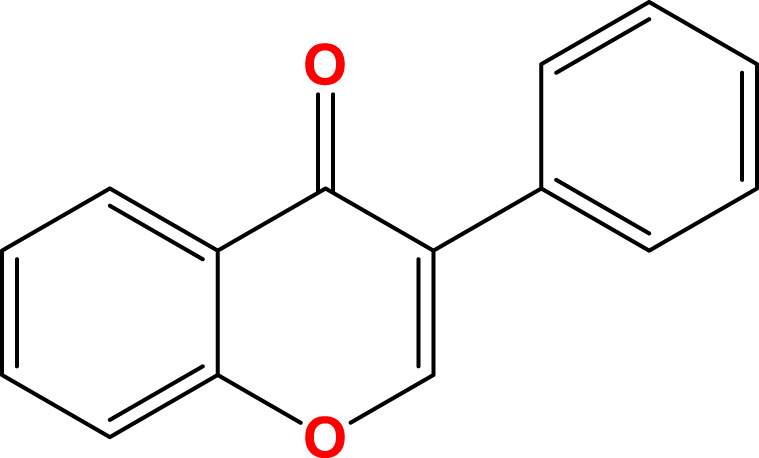 3-phenylchromen-4-one skeleton	Soybean and soybean products, Red clover	Genistein, Daidzein, Equol	Gómez-Zorita et al. (2020), Křížová et al. (2019), Yamagata and Yamori (2021)
Anthocyanidins	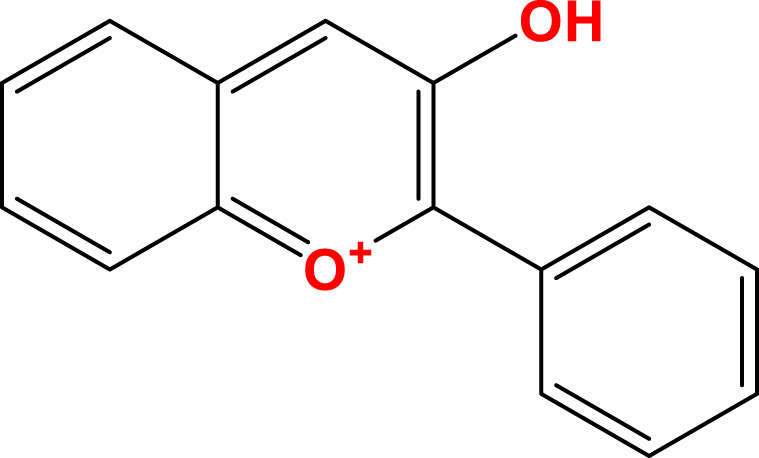 Anthocyanins are glycosides of polyhydroxy and polymethoxy derivatives	Flowers, Fruits, Seeds, Plant leaves	Cyanidin, Delphinidin, Malvidin	de Sousa Moraes et al. (2019), Lee et al. (2017), Wallace and Giusti (2015)
Chalcones (1,3-diaryl-2-propen-1-ones)	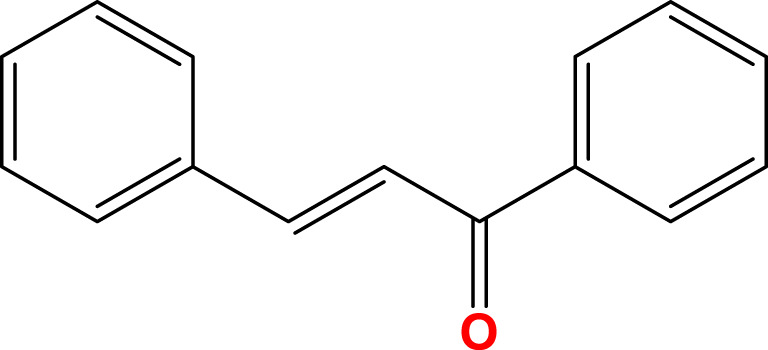 Open-chain flavonoids containing up to three modified or unmodified C5, C10 and C15 olefin molecules in the A and B rings	Fruits, Vegetables, Teas	Isoliquiritigenin, Isobavachalcone, Xanthohumol	Constantinescu and Lungu (2021), WalyEldeen et al. (2023)

Recent studies highlight the central role of the gut-liver axis in MAFLD pathogenesis through oxidative stress, inflammation, and lipid dysregulation (De Cól et al., 2024; Fianchi et al., 2021; Hu Y. et al., 2025; Peng et al., 2024). This communication system between the gut microbiota, the gastrointestinal tract, and the liver underpins MAFLD development (Kuziel et al., 2025; Luo et al., 2023; Martín-Mateos and Albillos, 2021). For instance, microbiota dysbiosis can cause translocation of microbial products like lipopolysaccharides (LPS), triggering endotoxemia, systemic inflammation, and liver injury (Luo et al., 2023; Peiseler et al., 2022). Microbial metabolites also have emerged as important modulators in the development of hepatic steatosis. Altered bile acid (BA) profile resulting from microbiome imbalances contributes to hepatic steatosis by affecting both BA signalling and lipid metabolism, reinforcing the gut-liver interaction and advancing the pathogenesis of MAFLD (De Cól et al., 2024; Luo et al., 2023).Short-chain fatty acids (SCFAs), produced by gut microbiota fermentation, enhance the intestinal barrier and modulate liver fatty acid (FA) metabolism. SCFAs decrease hepatic triglyceride accumulation, improve insulin sensitivity, and reduce inflammation, mitigating MAFLD progression (Fianchi et al., 2021; Barber et al., 2023).

Metabolite of dietary flavonoids by gut bacteria, such as S-equol, which improve liver health by modulating oxidative stress and inflammation (Rao et al., 2021; Qi et al., 2025). The interaction between gut metabolism and flavonoid bioavailability also provides an important basis to understands flavonoid’s role to intervene MAFLD development (Cheng et al., 2024). Studies have shown that gut dysbiosis, which is often associated with MAFLD, can impair the metabolic conversion of flavonoids, reducing their protective effects on liver health (Long et al., 2024; Fang et al., 2024).

Given the structural diversity of flavonoid and their complex interaction with gut microbial community, this review aims to summarize how flavonoids target the gut-liver axis to mitigate MAFLD. It emphasizes microbiota-dependent and -independent mechanisms to provide novel therapeutic strategies for MAFLD management through medical or dietary interventions.

## 2 Flavonoid, and their metabolism by gut microbiota

Flavonoids represent the principal bioactive constituents in a wide array of medicinal plants and have been employed in the management of numerous diseases, including MAFLD. Owing to their multi-targeted biological activity, minimal toxicity, and dietary origin, flavonoids have garnered increasing scientific interest for their therapeutic potential in mitigating MAFLD. This growing interest is substantiated by findings from evidence-based medicine derived from population-level data. For instance, analyses of the National Health and Nutrition Examination Survey (NHANES) database have highlighted the hepatoprotective properties of dietary flavonoids in reducing the risk of MAFLD (Tong et al., 2022). Moreover, a meta-analysis of randomised controlled trials has indicated that flavonoids—such as quercetin, epicatechin, naringenin, apigenin, among others—ameliorate MAFLD by enhancing hepatic metabolic function, attenuating inflammatory responses, and modulating gut microbiota composition (Li et al., 2023b).

**FIGURE 2:**
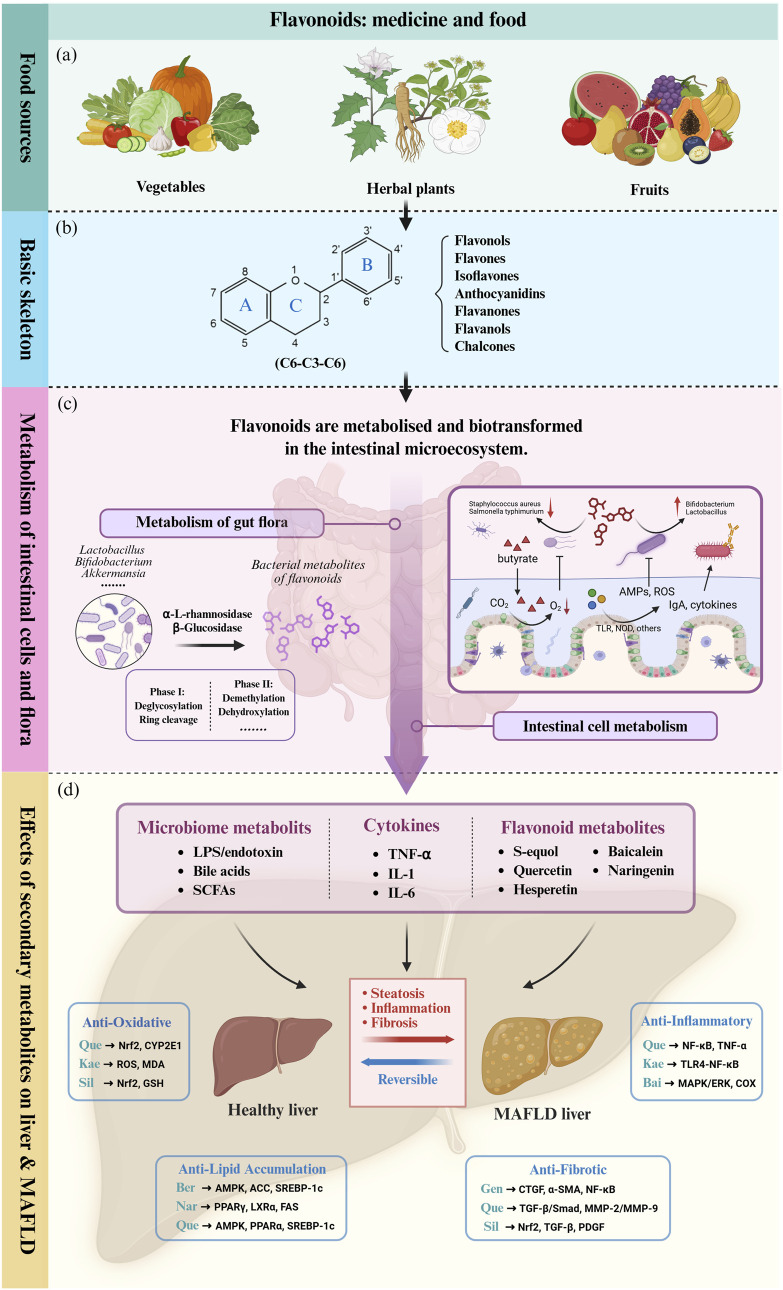
Flavonoids influence liver metabolism and MALFD progression directly or indirectly through the gut-liver axis. **(a)** Flavonoids are widely distributed in the natural diet and are abundant in various medicinal plants, vegetables and fruits. **(b)** The basic skeletal structure and seven subclasses of flavonoids. **(c)** The intestinal microenvironment and its role in flavonoid metabolism and biotransformation. **(d)** Flavonoids and intestinal secondary metabolites ultimately act on the liver and affect pathophysiological processes of MAFLD such as hepatic inflammatory cascade, oxidative stress and lipid accumulation through complex molecular mechanisms (In the image, upstream and downstream pathways use the same color to distinguish signaling pathways driven by different bioactive molecules). TLR: Toll-like Receptor. TLR4: Toll-like Receptor 4. NOD: Nucleotide-binding Oligomerization Domain-containing protein. ROS: Reactive Oxygen Species. LPS: Lipopolysaccharide. SCFA: Short-Chain Fatty Acid. TNF-α: Tumor Necrosis Factor-alpha. IL-1: Interleukin-1. IL-6: Interleukin-6. Nrf-2: Nuclear Factor Erythroid 2-related Factor 2. CYP2E1: Cytochrome P450 Family 2 Subfamily E Member 1. MDA: Malondialdehyde. GSH: Glutathione. AMPK: AMP-activated Protein Kinase. ACC: Acetyl-CoA Carboxylase. SREBP-1c: Sterol Regulatory Element-binding Protein 1c. PPARα: Peroxisome Proliferator-activated Receptor Alpha. PPARγ: Peroxisome Proliferator-activated Receptor Gamma. LXRα: Liver X Receptor Alpha. FAS: Fatty Acid Synthase. CTGF: Connective Tissue Growth Factor. α-SMA: Alpha-Smooth Muscle Actin. NF-κB: Nuclear Factor-kappa B. TGF-β: Transforming Growth Factor-beta. Smad: Homolog of the *Caenorhabditis elegans* protein SMA and *Drosophila* protein MAD. MMP-2: Matrix Metalloproteinase-2. MMP-9: Matrix Metalloproteinase-9. MAPK: Mitogen-Activated Protein Kinase. COX: Cyclooxygenase. ERK: Extracellular Regulated Protein Kinase. ApoB: Apolipoprotein B. Bid: BH3 interacting-domain death agonist. SIRT1: Sirtuin 1. UQ: Ubiquinone. Que: Quercetin. Kae: Kaempferol. Bai: Baicalein. Sil: Silymarin. Gen: Genistein. Nar: Naringenin. Ber: Berberine. Original image drawn for this review using Biorender software.

Actually, the intricate interplay between flavonoids and gut microbiota constitutes a bidirectional relationship that profoundly influences host physiology. Emerging evidence highlights the gut microbiome’s role in modulating flavonoid bioavailability and bioactivity, while flavonoids reciprocally reshape microbial ecology and metabolic output (Murota et al., 2018; Shabbir et al., 2021; Pei et al., 2020). This dynamic interaction forms a critical axis for understanding the therapeutic potential of flavonoids in metabolic diseases, including MAFLD (Figure 2), as will be elaborated upon in subsequent sections.

### 2.1 Introduction of diverse flavonoid structures

Flavonoids are a diverse group of plant-derived polyphenolic compounds, characterized by a common 15-carbon skeleton comprising two aromatic rings (A and B) and a heterocyclic ring (C), forming a C6–C3–C6 structure (Kumar and Pandey, 2013; Latos-Brozio and Masek, 2019). This core structure serves as the foundation of flavonoids subclasses, each distinguished by specific modifications that influence their biological activities and physicochemical properties (Dias et al., 2021; Shen et al., 2022). Through extensive modification, it yields >8,000 species across seven major subclasses (Figure 1): flavones characterized by a 2-phenylchromen-4-one backbone (e.g., luteolin, apigenin), flavonols which are 3-hydroxylated flavones (e.g., quercetin, kaempferol) flavanones that lack the C2 = C3 double bond (e.g., naringenin, hesperetin), flavanols that lack the C2 = C3 double bond and C4 = O carboxyl group, but have C3-hydroxyl group (e.g., catechins, epicatechins), isoflavones with B ring attached to C3 (e.g., genistein, daidzein), anthocyanidins with C1 oxonium ion and C3-hydroxyl group (e.g., cyanidin, delphinidin), chalcones which are open-chain flavonoids serving as precursors in flavonoid biosynthesis (e.g., phloretin, isoliquiritigenin) (Shen et al., 2022; Kumar and Pandey, 2013).

Beyond these core structures, flavonoids undergo various modifications through hydroxylation, methylation (mainly O-methylation), glycosylation, and polymerization, that enhance their structural diversity and functional properties (Wang et al., 2020). Additional hydroxylation increases polarity and antioxidant capacity, while methoxylation enhances lipophilicity and membrane permeability, facilitating absorption and bioavailability. Glycosylation, the attachment of sugar moieties, improves water solubility and stability, and can substantially influence their bioactivity. Polymerization leads to the formation of oligomeric compounds, such as proanthocyanidins, which exhibit unique antioxidant properties (Cao et al., 2015).

These structural features and modifications not only define the chemical nature of flavonoids but also underpin their vast array of biological activities, including antioxidant, anti-inflammatory, and anticancer properties. Understanding the structural diversity of flavonoids is crucial for elucidating their mechanisms of action and potential therapeutic applications.

### 2.2 Flavonoid metabolism by gut microbiota

#### 2.2.1 Two-stage microbial biotransformation of flavonoids

The gut microbiota mediates the structural modification and metabolism of dietary flavonoids through a complex enzymatic network, including phase I (deglycosylation, ring cleavage) and phase II (demethylation, dehydroxylation) reactions (Loo et al., 2020; Liu C. et al., 2024; Tian et al., 2019). In the first stage, key bacterial enzymes (e.g., β-glucosidase, α-rhamnosidase, and UDP-glucuronosyltransferases) and host intestinal lactase convert β-glycosylated flavonoids to more easily absorbed aglycone compounds (Loo et al., 2020; Liu Z. et al., 2021; Wang et al., 2023). Following absorption, these flavonoid aglycones undergo further biotransformation through phase II reactions. These reactions, predominantly mediated by the gut microbiota, include critical modifications such as glycosylation, hydroxylation, O-methylation and depolymerization. Microbial metabolites derived from flavonoids exhibit different bioactivity compared to their parent compounds. These metabolites often survive hepatic first-pass metabolism, achieving higher systemic concentrations and longer half-lives (Liu C. et al., 2024).

#### 2.2.2 Impact of flavonoid structure on microbial metabolism

The structure of flavonoids not only dictates their biological activities and target interactions but also profoundly influences their metabolic fate within the gut microbiota. We summarised the metabolic transformations of selected flavonoids based on gut flora and their targets of action regarding MAFLD treatment (Table 2).

**TABLE 2:** Gut microbiota-mediated flavonoid metabolism and its molecular mechanisms in MAFLD.

Flavonoid substrate	Gut microbiota metabolite	Reaction (enzyme)	Microbial species/Strain	Molecular targets/Pathways	Effects on liver and MAFLD	References
Rutin	Quercetin	Deglycosylation (α-L-rhamnosidase, β-Glucosidase)	*Bifidobacterium* spp.	PPARα^*^, SREBP-1c^#^, NF-κB^#^	Reduces hepatic lipid accumulation and inflammation via activation of FA β-oxidation, suppression of lipogenesis, and enhancements of mitochondrial biogenesis	Shi et al. (2022), Ferreira-Lazarte et al. (2021), Feng et al. (2021)
			*Clostridium orbiscindens, Lactobacillus* spp.	Nrf2^*^, HO-1^*^, NQO1^*^		
			*Bacteroides* spp.*, Eubacterium* spp.	AMPK^*^, PPARγ^*^		
Daidzein	S-Equol	Reductive ring cleavage (Daidzein reductase)	*Slackia isoflavoniconvertens, Eggerthella lenta*	ERβ^*^, PPARα^*^, CPT1A^*^, SREBP-1c^#^	Suppresses hepatic lipogenesis and enhances FA β-oxidation via estrogen receptor signalling	Kiyama (2023), Huang et al. (2019), Kumari et al. (2024), Křížová et al. (2019), Yamagata and Yamori (2021), Zhang et al. (2024b)
Genistein	Equol	Reductive ring cleavage (Daidzein reductase)	*Slackia isoflavoniconvertens*	ERβ^*^, Wnt/β-catenin^#^	Inhibits hepatic stellate cell activation and fibrosis by targeting Wnt signalling	Kiyama (2023), Kuziel et al. (2025), Farhat et al. (2023)
Hesperidin	Hesperetin	Deglycosylation (β-glucosidase)	*Bacteroides* spp.	FXR^*^, SREBP-1c^#^, PPARγ/Adiponectin^*^	Reduces hepatic triglycerides by activating farnesoid X receptor and suppressing lipogenesis, and improves insulin sensitivity	Xu et al. (2024), Barreca et al. (2017)
Apigenin	p-Hydroxyphenylacetic acid	Ring cleavage (Flavone reductase)	*Clostridium orbiscindens*	LXRα^*^, ABCA1/ABCG1^*^	Enhances cholesterol efflux and reverse cholesterol transport in macrophages	Jeong et al. (2022), Hostetler et al. (2017), Li and Somerset (2018)
Luteolin	3-Hydroxyphenylacetic acid	Ring cleavage (Flavone reductase)	*Clostridium orbiscindens*	NF-κB^#^, NLRP3^#^	Suppresses hepatic inflammation by inhibiting NLRP3 inflammasome activation	Li et al. (2021a), Yang et al. (2025), Jeong et al. (2022), Hostetler et al. (2017), Liu et al. (2021b)
	Protocatechuic acid		*Lactobacillus* spp.*, Bacteroides* spp.	PPARγ^*^, GLUT4^*^		
Myricetin	Pyrogallol	Deglycosylation, Ring Cleavage (β-glucosidase, Ring-cleaving dioxygenase)	*Lactobacillus* spp.*,* *Bacteroides* spp.	Nrf2^*^, HO-1^*^	Enhances antioxidant defense and reduces oxidative stress in hepatocytes	Dias et al. (2021), Fan et al. (2018), Popiolek-Kalisz and Fornal (2022), Wendlocha et al. (2024), Sun et al. (2021)
Catechin	5-(3,4-Dihydroxyphenyl)-γ-valerolactone	Ring cleavage (Catechin dioxygenase)	*Eubacterium* spp.*,* *Lactobacillus* spp.	AMPK^*^, SIRT1^*^	Activates energy-sensing pathways and improves mitochondrial function	Shabbir et al. (2021), Liu et al. (2024a), Talib et al. (2024), Dey et al. (2020), Huang et al. (2020)
Baicalin	Baicalein	Hydrolysis (β-glucuronidase)	*Lactobacillus* spp.	TLR4/NF-κB^#^, Nrf2^*^	Reduces hepatic oxidative stress and inflammation via Nrf2 activation	Lin et al. (2022), Peng et al. (2021), Yu et al. (2024), Hu et al. (2021)
Naringin	Naringenin	Hydrolysis (β-glucosidase)	*Clostridium* spp.	FXR/TGR5^*^, LXRα^*^	Improves bile acid homeostasis and cholesterol metabolism	Ferreira-Lazarte et al. (2021), Feng et al. (2020)
Quercetin-3-O-glucoside	Quercetin aglycone	Deglycosylation (β-glucosidase)	*Akkermansia muciniphila*	SIRT1^*^, Nrf2^*^, NF-κB^#^	Reduces LPS-induced hepatic inflammation and insulin resistance	Ferreira-Lazarte et al. (2021)

“*” Indicates that the target/pathway is activated by flavonoids. “#” indicates that the target/pathway is inhibited by flavonoids.

##### 2.2.2.1 Glycosylation

Glycosylation significantly impacts flavonoid bioavailability and target engagement (Zeng et al., 2020; Shi et al., 2022). C-linked glycosides (e.g., vitexin), resistant to hydrolysis by mammalian digestive enzymes, require microbial glycoside hydrolases (e.g., C-glycoside hydrolases contained in *Enterococcus faecalis*) for deglycosylation (Braune and Blaut, 2011). The resulting aglycones can directly interact with cellular targets, including nuclear receptors such as ER, FXR (Tian et al., 2019; Kiriyama et al., 2024). In addition, O-glycosylated flavonoids like rutin (quercetin-3-O-rutinoside) are hydrolyzed by intestinal α-L-rhamnosidases (e.g., from *Bifidobacterium* spp. and *Lactobacillus*), generating quercetin aglycone (Murota et al., 2018; Liu C. et al., 2024; Liu Z. et al., 2021; Fan et al., 2018). Flavonols with 3-O-glycone are preferentially hydrolyzed by *Lactobacillus* strains, which express β-glucosidases (Wang et al., 2023; Chen-Chen et al., 2024; Li B. et al., 2021; Zhu et al., 2024). Reciprocally, unique glycan constituents of flavonoids also affect microbial ecology. For instance, the rutin glycoside moiety acts as a prebiotic, selectively promoting *Bifidobacterium* growth and enhancing SCFA production, which in turn activates G-protein-coupled receptor 43 (GPR43) to improve hepatic energy metabolism (Shi et al., 2022; Ferreira-Lazarte et al., 2021).

##### 2.2.2.2 Hydroxylation

The number and position of hydroxyl groups on the flavonoid skeleton also dictate substrate specificity for microbial enzymes and target binding affinity. For instance, flavones possessing a 5,7-dihydroxy configuration, such as apigenin, are recognized substrates for gut microbes like *Flavonifractor plautii*. A key initial step in the intestinal catabolism of apigenin is the hydrogenation of its C2-C3 double bond. This reaction is catalysed by a flavone reductase (FLR) from *F. plautii*, which stereospecifically reduces apigenin to naringenin (a dihydroflavone), an essential intermediate for further degradation into phenolic acids (Yang et al., 2021). The activity of FLR is a critical gateway to the breakdown of dietary flavones.

Importantly, the specific hydroxylation pattern of a flavonoid can enable it to selectively modulate inflammatory responses. In the case of kaempferol, the 4′-hydroxyl group on its B-ring is crucial for the inhibition of the mitogen-activated protein kinase (MAPK) pathway, which suppresses the production of inflammatory cytokines by downregulating ERK1/2 phosphorylation (Niziński et al., 2025; Li N. et al., 2023). Furthermore, the 3,5,7-trihydroxy arrangement of kaempferol facilitates its interaction with Toll-like receptor 4 (TLR4), thereby blocking lipopolysaccharide (LPS)-induced NF-κB activation and attenuating liver inflammation (Niziński et al., 2025; Qu et al., 2021; Wu et al., 2024). Therefore, the anti-inflammatory properties of flavonoids can be modified by gut microbiome.

##### 2.2.2.3 O-methylation (methoxylation) and O-demethylation

Structural alkyl modifications of flavonoids, particularly O-methylation (forming methoxyl groups, -OCH_3_), critically determine their metabolic fate and bioactivity (Kim et al., 2014). Polymethoxyflavones (PMFs) like nobiletin, confers unique biological activities by enhancing membrane permeability and membrane receptor binding (Xu et al., 2024; Zhang et al., 2020). Increasing evidence highlights the significant impact of gut microbiota on flavonoid methylation status. Although direct microbe-mediated O-methylation of flavonoids has been less extensively studied, specific microbial enzymes, such as DnrK from *Streptomyces peucetius*, have been shown to perform O-methylation *in vitro* on various flavonoids, including apigenin and genistein, typically at the C7 hydroxyl group (Kim et al., 2007). This suggests a potential for similar activities within the complex gut microbiome, even if not yet fully characterized *in situ*.

Conversely, microbial O-demethylation of many methoxylated flavonoids is relatively well documented. For instance, PMFs often undergo extensive O-demethylation by gut bacteria, particularly at positions like C-3′ and C-4′ on the B ring (Cao et al., 2015; Braune and Blaut, 2016). This may exhibit different bioactivities, absorption profiles, and targets in the host compared to their parent compounds. For example, *Aspergillus niger* strains have been shown to regioselectively O-demethylate tangeretin and 3-hydroxytangeretin into their 4′-O-demethylated metabolites, demonstrating a microbial capacity for targeted demethylation similar to some mammalian P450 systems (Murota et al., 2018; Buisson et al., 2007).

##### 2.2.2.4 Depolymerization

Flavonoid polymers such as oligomeric proanthocyanidins, polymeric rutin, and condensation complexes of catechin exhibit significantly delayed microbial catabolism compared to their monomers (Latos-Brozio and Masek, 2019; Patanè et al., 2023). Specifically, crosslinked rutin shows a 5.6-fold prolonged intestinal retention time relative to its monomeric form due to reduced passive diffusion across enterocytes (Latos-Brozio and Masek, 2019). This kinetic property allows sustained release of bioactive metabolites in the distal colon (Liu C. et al., 2024; Shi et al., 2021). Due to their complex structures and high molecular weights, polymeric flavonoids like proanthocyanidins are poorly absorbed in the small intestine and a substantial portion reaches the colon largely intact (Braune and Blaut, 2016; Niwano et al., 2022). Here, the diverse enzymatic machinery of the gut microbiota plays a vital role in their breakdown. This microbial processing is initiated by depolymerization, a critical prerequisite for their biological activity where gut bacteria cleave the interflavan bonds (C-C and C-O-C linkages) holding the monomers together (Murota et al., 2018; Braune and Blaut, 2016). For instance, glycoside hydrolases from *Lactobacillus* and *Bacteroides* species target the glycosidic bonds in proanthocyanidins. These enzymes are induced by flavonoid exposure and show higher activity toward oligomers (DP 2–4) than high-molecular-weight polymers (Xiong et al., 2023). In addition, proanthocyanidin polymers (DP > 20) show limited depolymerization within the gastrointestinal tract, but their partial degradation by *Bifidobacterium* species produces metabolites (e.g., 5-(hydroxyphenyl)-γ-valerolactone) that modulate hepatic lipid metabolism via the gut-liver axis (Niwano et al., 2022; Shoji et al., 2023; Déprez et al., 2000). These differences in kinetics and bioactivity place the gut microbiota in a unique position in the gut-liver axis regulation of polymeric flavonoids.

However, despite the well-established enzymatic framework we outlined in 2.2, a critical translational gap remains between identifying microbial metabolic capabilities and confirming their physiological relevance in MAFLD. For instance, while bacterial β-glucosidases from *Lactobacillus* spp. are known to hydrolyze rutin to quercetin, and *F. plautii* can hydrogenate apigenin to naringenin, the functional outcomes of these transformations are often inferred rather than definitively proven. Meanwhile, the field suffers from significant quantification deficit, the inadequate characterization and quantification of the terminal active metabolites: despite knowing that microbial metabolites like equol exhibit potent bioactivities *in vitro* (e.g., activating AMPK or FXR), their actual concentrations achieved in human portal circulation or hepatic tissue following dietary flavonoid intake are scarcely measured. It is therefore plausible that many proposed mechanisms operate at pharmacologically irrelevant concentrations. If the intrinsic concentrations fall substantially below these thresholds, the proposed mechanisms and physiological significance of flavonoid-derived metabolites become questionable.

Furthermore, the immense inter-individual variability in gut microbiota composition means that the metabolic pathways detailed herein—such as the production of S-equol from daidzein by *Slackia isoflavoniconvertens*—may be absent or inefficient in a substantial proportion of the MAFLD population. Consequently, the promising effects observed in preclinical models may not consistently translate to human patients. Future research must prioritize absolute quantification of microbial flavonoid metabolites in human biospecimens using advanced techniques—such as targeted metabolomics, and *in vivo* imaging—to accurately quantify and spatially resolve the distribution of these metabolites in target tissues, thereby moving beyond correlative associations to establish causative links, and finally distinguish truly impactful metabolic pathways from mere observational curiosities.​​

### 2.3 Modulation of microbiota by flavonoid

As mentioned previously, flavonoids and gut microbiota engage in dynamic, reciprocal interactions that transcend mere metabolism, influencing both microbial composition and host physiology. Many flavonoids exert prebiotic-like effects by selectively enriching beneficial symbionts while suppressing pathobionts (Shabbir et al., 2021; Zhu et al., 2024; Naudhani et al., 2021).

For instance, theabrownin and quercetin increases symbionts *Bifidobacterium* and *Akkermansia muciniphila* abundances, while concurrently decrease the abundance of detrimental bacteria, such as *Proteobacteria*, *Bacteroides*, *Escherichia-Shigella*, and *Escherichia_coli* in murine models (Huang et al., 2019; Yuan et al., 2024). Importantly, these shifts in microbial community structure are often accompanied by significant alterations in overall microecological diversity. Studies have indeed reported that quercetin can modulate both alpha and beta diversity, leading to a more balanced and diverse gut microbiota composition, which is generally associated with improved gut health outcomes (e.g., increased Shannon, Simpson and Chao1 indices for alpha diversity, and PCoA and weighted UniFrac tree analysis for beta diversity) (Mi et al., 2022; Li et al., 2024a). Furthermore, these microecological changes translate into tangible physiological improvements in the host. Quercetin has been shown to reverse gut microbiota dysbiosis and inhibit the endotoxemia-mediated TLR-4 pathway, thereby ameliorating lipid metabolism abnormalities and mitigating systemic inflammation (Yuan et al., 2024; Porras et al., 2017; Cai et al., 2024). Compared to quercetin, isoquercetin exhibits a stronger ability to improve the MAFLD phenotype in mice induced by high-fat diet-fed (HFD). Isoquercetin significantly increases the abundance of *Bifidobacterium*, *Lactobacillus*, and *Akkermansia*, leading to the production of more SCFAs and indole metabolites, which leads to a reduction in hepatic steatosis in HFD mice (Tan et al., 2018). Similarly, luteolin and kaempferol increase the *Firmicutes*/*Bacteroidetes* ratio through upregulation of mucin-degrading *A. muciniphila*, which enhances gut barrier integrity (Li B. et al., 2021; Qu et al., 2021). This effect is partially mediated by flavonoid-induced inhibition of bile salt hydrolases (BSH), which alters intraluminal BA profiles. Specifically, BSH inhibition generally leads to an increase in conjugated BAs and a decrease in deconjugated BAs, thereby creating a microenvironment less favourable for certain pathogenic bacteria and more conducive to the growth of probiotic bacteria (Xu et al., 2024; Collins et al., 2023; Sayin et al., 2013).

Anthocyanins, which derived from black rice and blackcurrant (*Ribes nigrum* L.), was shown to enhance the proportion of SCFA-producing microbiota by promoting the growth of *Lactobacillus*, *Bifidobacterium*, and *A. muciniphila*, while suppressing pro-inflammatory pathogenic taxa such as *Helicobacter* and *Desulfovibrio.* Concurrently, these anthocyanins activate the PPARα, FXR, and AMPK signalling pathways and downregulate the expression of SREBP-1c, thereby contributing to improved hepatic lipid metabolism (Song et al., 2021a; Song et al., 2021b).

### 2.4 MAFLD evidence: based on the gut-liver axis

Accumulating clinical evidence positions flavonoids as promising therapeutics for MAFLD through microbiota-dependent mechanisms. Notably, fecal microbiota transplantation from flavonoid-treated mice recapitulates these metabolic benefits, confirming the functionality of flavonoid-trained microbial community (Fang et al., 2021). Moreover, flavonoids also mitigate MAFLD through multiple microbial-triggered pathways, including the inhibition of TLR4-NF-κB signalling to dampen hepatic inflammation (Qu et al., 2021; Porras et al., 2017; Huiru et al., 2023; Li et al., 2024b; Ting et al., 2022), activation of AMPK to promote FA β-oxidation (Song et al., 2021b; Li X. et al., 2021), and regulation of SCFA production to improve energy metabolism, among others (Kiriyama et al., 2024). These observations highlight the gut microbiota as a central hub through which flavonoids exert their hepatoprotective effects, underscoring the therapeutic potential of microbiota-targeted flavonoid interventions in MAFLD. Obviously, further exploration of the specific mechanisms is a great temptation for researchers in this field.

## 3 Mechanism of flavonoids action in MAFLD via the gut-liver axis

**FIGURE 3:**
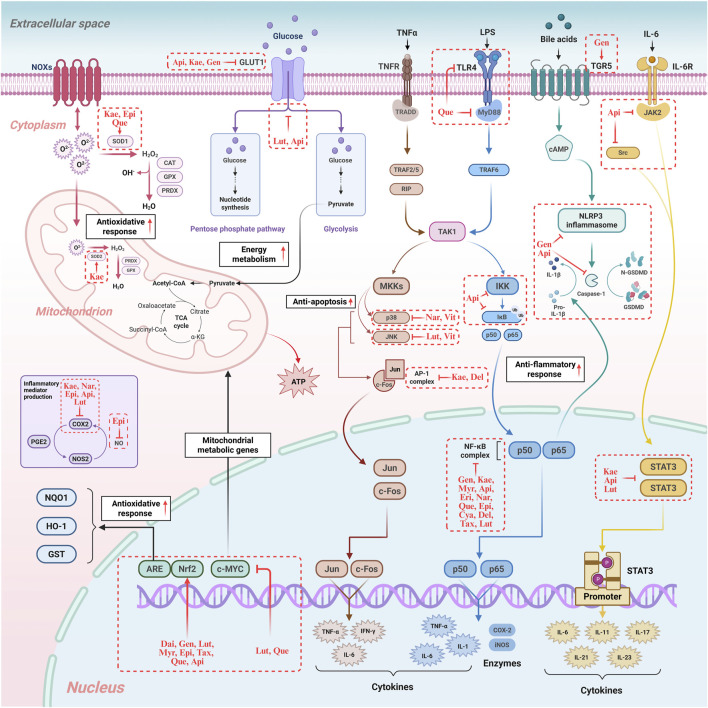
Regulatory mechanisms of flavonoids in hepatocyte oxidative stress, inflammation, immunity, and energy metabolism. The diagram illustrates how flavonoids modulate crucial signalling pathways, including those regulating oxidative stress (e.g., Nrf2 activation), inflammation (e.g., NF-κB inhibition), and energy metabolism (e.g., TCA cycle), highlighting their multi-target therapeutic potential. NOXS: NADPH Oxidases. SOD1: Superoxide Dismutase 1. SOD2: Superoxide Dismutase 2. CAT: Catalase. GPX: Glutathione Peroxidase. PRDX: Peroxiredoxin. GLUT1: Glucose Transporter 1. ATP: Adenosine Triphosphate. PGE2: Prostaglandin E2. COX2: Cyclooxygenase-2. iNOS: Inducible Nitric Oxide Synthase. NOS2: Nitric Oxide Synthase 2. NQO1: NAD(P)H Quinone Dehydrogenase 1. IDH1: Isocitrate Dehydrogenase 1. ME1: Malic Enzyme 1. MAF: Musculoaponeurotic Fibrosarcoma Proteins. Nrf2: Nuclear Factor Erythroid 2-related Factor 2. c-MYC: cellular myelocytomatosis viral oncogene homolog. TNFR: Tumor Necrosis Factor Receptor. TRADD: TNF Receptor-Associated Death Domain. TRAF2/5: TNF Receptor-Associated Factor 2/5. TRAF6: TNF Receptor-Associated Factor 6. RIP: Receptor-Interacting Protein. TAK1: TGF-β-Activated Kinase 1. MKKs: Mitogen-Activated Protein Kinase Kinases. p38: p38 Mitogen-Activated Protein Kinase. p50: NF-κB subunit p50. p65: NF-κB subunit p65. JNK: c-Jun N-terminal Kinase. c-Fos: FBJ murine osteosarcoma viral oncogene homolog. Jun: Transcription factor Jun. AP-1: Activator Protein-1. TNF-α: Tumor Necrosis Factor-alpha. IFN-γ: Interferon-gamma. IL-1: Interleukin-1. IL-1β: Interleukin-1 beta. IL-6: Interleukin-6. IL-11: Interleukin-11. IL-17: Interleukin-17. IL-21: Interleukin-21. IL-23: Interleukin-23. LPS: Lipopolysaccharide. TLR4: Toll-like Receptor 4. MyD88: Myeloid Differentiation Primary Response 88. IKK: IκB Kinase. IκB: Inhibitor of κB. NF-κB: Nuclear Factor-kappa B. TGR5: G Protein-coupled Bile Acid Receptor 1. cAMP: Cyclic Adenosine Monophosphate. NLRP3: NLR Family Pyrin Domain Containing 3. GSDMD: Gasdermin D. IL-6R: Interleukin-6 Receptor. JAK2: Janus Kinase 2. Src: Proto-oncogene tyrosine-protein kinase Src. STAT3: Signal Transducer and Activator of Transcription 3. Kae: Kaempferol. Epi: Epigallocatechin. Que: Quercetin. Api: Apigenin. Gen: Genistein. Lut: Luteolin. Nar: Naringenin. Dai: Daidzein. Myr: Myricetin. Tax: Taxifolin. Eri: Eriodictyol. Cya: Cyanidin. Del: Delphinidin. Vit: Vitexin. Original image drawn for this review using Biorender software.

In recent years, a growing number of meta-analyses and systematic reviews with high-quality of evidence-based medicine evidence have pointed to flavonoid supplementation as a promising pharmacological option for the management of MAFLD and its associated complications (Li et al., 2023b; Liu H. et al., 2024). Their therapeutic efficacy is largely attributed to their ability to modulate the gut-liver axis: flavonoids can act directly on the liver after modification by the gut microbiota, or they can work synergistically with microbial metabolites to accomplish cooperative signalling (Figure 3). These findings are supported by a large body of animal studies, which have elucidated the diverse mechanisms by which flavonoids exert their therapeutic effects on MAFLD (Table 3). However, these promising results must be interpreted with caution due to significant translational limitations inherent in current animal models. The widely used HFD model effectively recapitulates hepatic steatosis and insulin resistance, but often fails to fully replicate the profound inflammatory component and fibrotic progression characteristic of human MASH. Conversely, while the methionine-choline deficient (MCD) diet model rapidly induces steatohepatitis and fibrosis, its accompanying weight loss paradoxically contradicts the typical obese phenotype observed in most human MASH patients. Furthermore, the pharmacological doses employed in many animal studies (e.g., baicalein at 400 mg/kg/day) vastly exceed achievable human dietary intake levels—when converted to human equivalent doses, these doses fall far beyond reasonable supplementation ranges, and raise legitimate concerns about potential toxicity. Therefore, considerable challenges still remain in translating these findings into clinically relevant, dietary achievable interventions for human MAFLD.

**TABLE 3:** Animal experiments related to the treatment of MAFLD.

Flavonoid name	Disease treated	Animal model/Number	Intervention dosage	Key results	Mechanisms	References
Quercetin	NAFLD	C57BL/6J mice (n = 40)	Quercetin 0.05%/day, oral, 16 weeks	Significantly reduced hepatic lipid accumulation, improved metabolic markers and gut microbiota dysbiosis	Quercetin modulates intestinal microbiota imbalance and relates gut-liver axis activation. It reduces the *Firmicutes/Bacteroidetes* ratio, inhibits endotoxemia-mediated TLR-4/NF-κB signaling, and upregulates lipid β-oxidation genes like PPAR-α and CPT-1a	Porras et al. (2017)
Silymarin	MASLD	ICR mice (n = 50)	30 or 80 mg/kg/day, oral, 8 weeks	Attenuated liver inflammation and fibrosis, and gut microbiota modulation	Silymarin regulates gut microbiota homeostasis and the TLR4/NF-κB signaling pathway. It specifically targets the FXR protein through the microbial metabolite 7-keto-deoxycholic acid (7-KDCA), which acts as an FXR antagonist	Yi et al. (2024)
Baicalein	Metabolic disorder	C57BL/6J mice (n = 80)	400 mg/kg/day, oral, 29 weeks	Selectively activated AMPK_α2_, ameliorating insulin resistance and lipid abnormalities	Baicalein selectively activates AMPK_α2_ which leads to the inhibition of the MAPKs pathway, the blocking of lipid synthesis by inhibiting SERBP-1c and PPARγ, and the enhancement of fatty acid oxidation	Pu et al. (2012)
Baicalein	NAFLD	C57BL/6N mice (n = 50)	100 or 200 mg/kg/day, oral, 5 weeks	Suppressed hepatic steatosis and oxidative stress and altered gut microbiota composition	Baicalein remodels the gut microbiota structure and affects lipid metabolism in the liver by regulating the gut-liver axis	Li et al. (2022)
Quercetin + *Akkermansia*	Obesity and NAFLD	Wistar rats (n = 60)	Quercetin 37.5 mg/kg/day, *Akkermansia* 2 × 10^8^ CFU oral, 10 weeks	Synergistically reshaped gut microbiota and modulated bile acid metabolism	The synbiotic combination reshapes the gut microbiota, modulates bile acid metabolism, modulates liver lipogenesis and increases the plasma levels of unconjugated hydrophilic bile acids	Juárez-Fernández et al. (2021)

### 3.1 Direct hepatic effects of microbiota-modified flavonoids

The human gut-liver axis is increasingly recognized as a key regulator of hepatic metabolic health, and in addition to its direct involvement in hepatic physiology (e.g., BA metabolism), it can affect the liver through its ability to alter bioactive compounds. The gut microbiota converts dietary flavonoids into metabolites that directly modulate hepatic signalling. This is expected to address the lipid dysregulation, oxidative stress, and inflammatory response that are core pathological features of MAFLD.

#### 3.1.1 Regulation of lipid metabolism

Flavonoids can inhibit *de novo* lipogenesis via key transcription factors like Sterol Regulatory Element-Binding Protein 1c (SREBP-1c) and lipogenic enzymes. In some cases, they also promote fatty acid oxidation by activating master regulators like PPARα, rate-limiting enzymes such as Carnitine Palmitoyltransferase 1 (CPT1), and enhancing alternative oxidation pathways like ω-oxidation. In brief, flavonoids contribute to systemic remodelling of lipid, improving circulating lipid profiles and influencing BA synthesis and excretion.

##### 3.1.1.1 Inhibition of hepatic lipogenesis

The synthesis process of new fatty acids and triglycerides in the liver is known as *de novo* lipogenesis (DNL), which is a critical contributor to hepatic steatosis in MAFLD (Gnoni et al., 2022; Ponugoti et al., 2010). A primary molecular target in this process is SREBP-1c, a master transcriptional regulator of hepatic lipogenesis (Ponugoti et al., 2010; Yoon et al., 2009). Flavonoids such as quercetin and baicalin have been shown to significantly reduce the expression of SREBP-1c and lipogenic genes (Gnoni et al., 2022; Jiang L. et al., 2025). Acetyl-CoA Carboxylase (ACC) and Fatty Acid Synthase (FASN) are key rate-limiting enzymes in fatty acid synthesis (Yoon et al., 2009; Jiang L. et al., 2025; Mu et al., 2020; Dong et al., 2025). Quercetin exerts its anti-lipogenic effect by phosphorylating Acetyl-CoA Carboxylase Alpha (ACACA), a key player that catalyses the committing step in the DNL pathway (Gnoni et al., 2022; Wan et al., 2025). Furthermore, baicalin suppresses DNL by inhibiting the AMPK/acetyl-CoA carboxylase pathway and downregulating FASN (Dai et al., 2018). Licorice chalcone and luteolin also inhibit adipogenesis by activating the Sirtuin1/AMPK pathway (Tan et al., 2022). The consistent targeting of SREBP-1c, ACC, and FASN by various flavonoids indicates a convergent therapeutic strategy to suppress the core DNL pathway. However, the distinct upstream mechanisms, such as quercetin’s action on the ACACA/AMPK/PP2A axis versus licorice chalcone/luteolin’s SIRT1/AMPK activation, reveal diverse molecular effect points to achieve this common outcome.

##### 3.1.1.2 Promotion of hepatic fatty acid oxidation

Flavonoids enhance the breakdown of fatty acids for energy, thereby reducing intrahepatic fat levels (Aneta et al., 2024; Ipsen et al., 2018). Fatty acid oxidation (FAO), which is crucial for maintaining lipid homeostasis (Ipsen et al., 2018), is mainly regulated by hepatic Peroxisome Proliferator-Activated Receptor alpha (PPARα), particularly during fasting, that orchestrate the transcription of numerous FAO genes (Ipsen et al., 2018; Silva and Peixoto, 2018). Flavonoids (including quercetin, naringenin and baicalin) have been shown to ameliorate hepatic fat accumulation by targeting PPARα/γ (Jiang L. et al., 2025; Dong et al., 2025; Dai et al., 2018; Zhao et al., 2023).

Another critical molecular target is CPT1, a rate-limiting enzyme that facilitates lipid influx into mitochondria for FAO (Jiang L. et al., 2025; Dai et al., 2018). Baicalin directly activates hepatic CPT1, accelerating this process (Dai et al., 2018). Quercetin enhances CPT1A expression, and increases hepatic lipid ω-oxidation, leading to lowered circulating lipid levels (Jiang L. et al., 2025; Hoek-van den Hil et al., 2013). The consistent promotion of FAO by flavonoids via PPARα and CPT1 directly addresses the insufficiency of compensatory FAO often observed in MAFLD, which can otherwise lead to oxidative stress and disease progression (Ipsen et al., 2018). The ability of quercetin to increase ω-oxidation provides an additional, distinct pathway for fatty acid disposal, which is particularly important when mitochondrial β-oxidation is overwhelmed or compromised. This suggests that flavonoids may protect against lipotoxicity not merely by reducing fat production, but by enhancing the liver’s capacity to safely process excess fatty acids, thereby preventing the “second hit” of oxidative stress and inflammation (Zhang S. et al., 2025).

#### 3.1.2 Regulation of BA Enterohepatic metabolism

Flavonoids act on FXR in the liver and intestine to regulate BA synthesis, excretion and reabsorption. This modulation of BA metabolism is one of the key axes influencing the pathological processes associated with MAFLD. By inhibiting intestinal FXR signalling, compounds like theabrownin from Pu-erh tea increase hepatic BA synthesis and fecal excretion, reducing hepatic cholesterol accumulation (Huang et al., 2019; Liang et al., 2024). Quercetin, a paradigmatic flavonol which alleviates hepatic steatosis in HFD mice, maintains lipid homeostasis and attenuates hepatic fat accumulation mainly by regulating intestinal BA metabolism and activating FXR and TGR5 in the liver (Yuan et al., 2024; Porras et al., 2017; Cai et al., 2024). S-equol, as previously noted, binds estrogen receptors with higher affinity than precursor daidzein, which not only exerts stronger anti-inflammatory and anticancer effects, (Farhat et al., 2023), but also activates hepatic FXR to regulate BA synthesis (Kumari et al., 2024). Moreover, naringenin enhances the production of secondary BAs (e.g., lithocholic acid) by inducing BSH activity in *Bacteroides ovatus*. These BAs activate FXR in the ileum and stimulate fibroblast growth factor 19 (FGF19) secretion (Katafuchi and Makishima, 2022). Hepatic FGF19 receptor (FGFR4) activation inhibits cytochrome P450 7A1 (CYP7A1), the rate-limiting enzyme in the synthesis of bile acids, and activates c-Jun N-terminal kinase (JNK), which phosphorylates and inhibits carbohydrate-responsive element-binding protein (ChREBP), reducing hepatic gluconeogenesis (Huang et al., 2019). At another metabolic node, flavonoids modulate BA metabolism by inhibiting BSH activity in *Clostridium* and *Bacteroides*, thereby increasing conjugated BA that antagonize intestinal FXR signalling (Zhang et al., 2020; Huang et al., 2019; Lin et al., 2022). Concurrently, altered BA profiles feedback on gut microbiota, suppressing BSH-positive pathogens and promoting beneficial bacteria (Xu et al., 2024). This bidirectional crosstalk targeting dysregulated BA metabolism that contributes to hepatic steatosis and inflammation holds key to the development of novel therapy for MAFLD (Xu et al., 2024; Huang et al., 2019).

#### 3.1.3 Modulation of oxidative stress

Various Flavonoids such as citrus-enriched naringenin exhibited potent anti-oxidative and anti-inflammatory properties. Demethylation of naringin by microorganisms produces naringenin, which undergoes further ring cleavage to produce phenolic acids (e.g., 4-hydroxyphenylacetic acid). These metabolites activate nuclear factor erythroid 2-related factor 2 (Nrf2), promoting its translocation to the nucleus and binding to antioxidant response elements (AREs) in the promoters of HO-1 (Heme Oxygenase-1), NQO1 (NAD(P)H: quinone oxidoreductase 1) and GCLC (glutamate-cysteine ligase catalytic subunit) (Dias et al., 2021). In HFD-induced MAFLD mice, naringenin supplementation increased hepatic glutathione (GSH) levels by 60%, reduced malondialdehyde (MDA) by 40%, and attenuated cytochrome P450 2E1 (CYP2E1)-mediated oxidative damage (Shi et al., 2022).

#### 3.1.4 Regulation of inflammatory via Kupffer cells (KCs)

Kupffer cells (KCs), the liver’s resident macrophages, their critical functions include recognizing and clearing foreign materials (such as bacterial products like LPS), and endogenous danger signals (Baffy, 2009). Activated KCs are significant contributors to hepatic inflammation and the progression of MAFLD to steatohepatitis. They release a variety of pro-inflammatory mediators, including cytokines, chemokines, and reactive oxygen species (ROS) (Baffy, 2009).

Flavonoids exert their anti-inflammatory effects on the liver through a dual approach. First, they can directly interact with KCs, as in case of bergamot polyphenols that shown to decrease hepatic inflammation by the expression of pro-inflammatory cytokines like interleukin-6 (IL-6) while increasing the anti-inflammatory cytokine IL-10 (Parafati et al., 2018). This effect correlated with fewer KCs and lower inflammatory foci scores in the liver, suggesting a direct immunomodulatory action (Parafati et al., 2018). Second, flavonoids modulate the inflammatory response via gut-liver axis by influencing the production and translocation of key microbial metabolites. In MAFLD, an impaired gut barrier allows the translocation of bacterial components like LPS from the intestinal lumen to the liver, that activate KCs via the Toll-like receptor 4 (TLR4) signalling pathway and subsequently the MyD88/NF-κB cascade, a central driver of pro-inflammatory gene expression. Flavonoids can directly inhibit this cascade, but they also have an indirect effect by modulating the gut microbiota to increase the production of anti-inflammatory metabolites, such as short-chain fatty acids (SCFAs) like butyrate. These SCFAs can then reach the liver via the portal vein and directly interact with KCs to suppress their inflammatory response. This multi-pronged approach is further facilitated by the fact that specific flavonoids, such as quercetin and luteolin, can modulate broader inflammatory networks. For example, they can affect the production of cytokines such as IL-17, which in turn influences KC activation and the subsequent inflammatory cascade (Jiang L. et al., 2025; Ma et al., 2020; Meng et al., 2012; Kelepouri et al., 2018).

#### 3.1.5 Regulation of inflammatory via hepatic T-cell

Beyond their influence on KCs, flavonoids exert a direct immunomodulatory effect on hepatic T-cells, which is crucial for managing liver inflammation and fibrosis. Specific flavonoids, such as curcumin and quercetin, have been shown to regulate T-cell activity by modulating key signalling pathways. For instance, curcumin suppresses T-cell activation by inhibiting calcium mobilization and the NFAT (Nuclear Factor of Activated T Cells) signalling pathway, leading to a dose-dependent reduction in the expression of pro-inflammatory cytokines like IL-2 and IFN-γ (Kliem et al., 2012). This effect is further supported by evidence that curcumin inhibits the proliferation of CD4^+^ T-cells (Kim et al., 2013). Similarly, quercetin has been found to modulate the balance between pro-inflammatory Th17 cells and anti-inflammatory regulatory T cells (Tregs), promoting an anti-inflammatory state within the liver (Jiang Z. et al., 2025).

Furthermore, flavonoids can induce apoptosis in activated T-cells, a mechanism essential for resolving inflammation. For example, baicalein selectively promotes apoptosis in activated lymphocytes, which helps to mitigate hepatitis by removing excessive inflammatory cells (Zhang et al., 2013). The anti-inflammatory actions of these compounds are often mediated by their ability to inhibit central signalling pathways such as NF-κB, MAPK, and the NLRP3 inflammasome, all of which are critical for T-cell activation and cytokine production (Jiang Z. et al., 2025; Martinez et al., 2019). These findings provide a cellular and molecular basis for how flavonoids can directly modulate hepatic immune responses, offering a promising therapeutic approach for MAFLD and other liver inflammatory conditions (Li et al., 2018; Wu and Wang, 2025).

#### 3.1.6 Regulation of fibrosis via hepatic stellate cells (HSCs)

Hepatic Stellate Cells (HSCs) play pivotal roles in the development and progression of liver fibrosis in chronic inflammation conditions such as in MAFLD. The activated HSCs are the primary producers of excessive extracellular matrix (ECM) proteins, which leads to the accumulation of fibrotic tissue (Zhang Y. et al., 2024). The activation of HSCs is driven by the dysregulation of multiple signalling pathways, including TGF-β/Smads, MAPK (ERK, JNK, p38), PI3K/AKT, Wnt, NF-κB, and AMPK (Zhang Y. et al., 2024). Transforming growth factor-beta (TGF-β) is a particularly potent activator of HSCs, promoting fibrosis through the Smad2/3 signalling pathway (Zhang Y. et al., 2024).

Flavonoids are recognized as promising natural compounds for alleviating or reversing hepatic fibrosis (Tauil et al., 2024). For example, quercetin, hydrolyzed from rutin by *Lactobacillus* β-glucosidases, can work with butyrate (a SCFA) to suppress TGF-β/Smad signalling in HSCs (Feng et al., 2021). Quercetin blocks TGF-β type I receptor (ALK5) phosphorylation, preventing Smad2/3 nuclear translocation and reducing COL1A1 and α-SMA transcription, while butyrate enhances this effect by inhibiting histone deacetylases (HDACs), increasing acetylation of the TGF-β promoter and reducing its expression (Wang S. et al., 2022). Genistein, a soy isoflavone, and urolithin A (derived from ellagitannins by *Enterococcus* and *Gordonella spp*) cooperate to inhibit the Wnt/β-catenin pathway, a key driver of HSCs activation (Farhat et al., 2023). Genistein binds to low-density lipoprotein receptor-related protein 5/6 (LRP5/6), blocking Wnt ligand binding and β-catenin stabilization, while urolithin A enhances this effect by promoting β-catenin ubiquitination and proteasomal degradation, reducing nuclear β-catenin levels and downstream fibrosis-related genes (e.g., CTGF, VEGFA) (Liu et al., 2025). Specific flavonoids have demonstrated clear anti-fibrotic effects.

#### 3.1.7 Flavonoids act on MAFLD via liver sinusoidal endothelial cells (LSECs)

Liver sinusoidal endothelial cells (LSECs) play a pivotal role in the development and progression of MAFLD. As a specialized cell type lining the liver sinusoids, LSECs are essential for maintaining liver homeostasis, regulating blood flow, and facilitating the bidirectional exchange of nutrients, hormones, and immune signals between the portal blood and hepatocytes. LSEC dysfunction, which can precede the development of inflammation and fibrosis, is now recognized as an early and critical event in MAFLD pathogenesis (Hammoutene et al., 2020; Velliou et al., 2023; Hammoutene and Rautou, 2019). This dysfunction is mechanistically characterized by several key changes, including the loss of fenestrations (defenestration) and the formation of a continuous basement membrane (capillarization). These structural alterations hinder the metabolic exchange between the bloodstream and hepatocytes, leading to lipotoxicity and a subsequent pro-inflammatory state. At the molecular level, this pathological process is exacerbated by a defect in endothelial autophagy, which has been observed in patients with non-alcoholic steatohepatitis (NASH) and contributes to inflammation and fibrosis by allowing the accumulation of damaged cellular components (Hammoutene et al., 2020). Furthermore, MAFLD-associated inflammation drives the overexpression of adhesion molecules, such as vascular cell adhesion molecule 1 (VCAM-1), on the surface of LSECs. This promotes the recruitment and adhesion of inflammatory cells, such as macrophages, to the liver, thereby accelerating the inflammatory cascade and the progression of fibrosis (Guo et al., 2022). By targeting these specific pathways—such as by protecting LSEC integrity, enhancing endothelial autophagy, or modulating adhesion molecule expression—flavonoids offer a promising therapeutic avenue for mitigating MAFLD progression.

### 3.2 Indirect effects of flavonoids on the MALFD via the gut-liver axis

In addition to direct effects on the liver, flavonoids also mediate synergistic signalling via intestinal epithelial cells and immune cells, which can significantly affect liver metabolism and disease progression. Such synergistic signalling networks amplify their effects on liver inflammation, fibrosis and metabolic homeostasis.

#### 3.2.1 Flavonoids act on MAFLD via intestinal epithelial cells (IECs)

Intestinal epithelial cells form a critical component of the gut barrier, regulating nutrient absorption and playing a significant role in metabolic signalling. Flavonoids have a multifaceted effect on these cells, particularly on regulating intestinal barrier function.

Dysregulation of the gut microbiota and subsequent intestinal barrier dysfunction are recognized contributors to the pathogenesis of MAFLD (Sun et al., 2025). Research shows that flavonoids can inhibit the loss of tight junction proteins such as ZO-1 and occludin, thereby improving intestinal barrier function. This protective action is attributed to the flavonoids’ ability to modulate the gut microbiota and an increased production of beneficial SCFAs (Dong et al., 2025; Aneta et al., 2024; Zhou et al., 2024). These SCFAs then exert protective effects on IECs, including the upregulation or maintenance of tight junction proteins like ZO-1 and occluding (Vancamelbeke and Vermeire, 2017). This sequence of events results in enhanced intestinal barrier function, a reduction in the translocation of bacterial endotoxins (such as LPS) to the liver, and ultimately, an attenuation of hepatic inflammation and MAFLD progression. This pathway highlights a crucial indirect mechanism by which flavonoids contribute to liver protection. For instance, total flavonoids derived from *Dracocephalum moldavica L.* have been shown to alleviate HFD rats by enhancing the intestinal barrier, alongside their anti-inflammatory and lipid metabolism-regulating effects (Sun et al., 2025).

#### 3.2.2 Flavonoids act on MAFLD via intestinal immune cells

The liver functions as a central immunological organ, and its susceptibility to inflammatory responses is particularly evident in chronic liver diseases such as MAFLD. The balance of intestinal T-cell responses, notably the Th17/Treg axis, further influences liver inflammation (Hammerich et al., 2011). Flavonoids exhibit immunomodulatory effects that can influence this delicate balance of intestinal T cell, impacting hepatic lipid inflammation.

The balance between Th17 cells and T regulatory (Treg) cells is crucial, as Tregs secrete anti-inflammatory cytokines and can mitigate the Th17 response, such as produce pro-inflammatory cytokines like IL-17 and IL-22 (Hammerich et al., 2011; Olveira et al., 2023; Abdelnabi et al., 2024; Pan et al., 2014). Levels of IL-17 have been correlated with the progression from MAFLD to steatohepatitis, cirrhosis, and even hepatocellular carcinoma (Hammerich et al., 2011; Olveira et al., 2023; Chackelevicius et al., 2016). In contrast, IL-22 is a pleiotropic cytokine that abrogates MASH-related inflammation and fibrosis development by inducing antioxidant and anti-apoptotic factors (Abdelnabi et al., 2024; Pan et al., 2014). This differentiation in function suggests that effective therapeutic strategies for MAFLD should aim to suppress the detrimental effects of IL-17 while potentially enhancing the beneficial actions of IL-22.

Previous studies have shown that flavonoids can significantly affect the release of IL-17 and IL-22 by regulating immune cell function and signalling pathways. For example, in the LPS-induced RAW 264.7 macrophage model, luteolin blocked the NF-κB signalling pathway, reduced the p65 binding activity in the promoter region of the IL-17A gene by 40%, led to downregulation of IL-17A mRNA expression, and inhibited the secretion of IL-17 (Gendrisch et al., 2021). In addition, a variety of flavonoids such as quercetin and naringenin have been shown to regulate the Th17/Treg cell ratio and affect IL-17/IL-22 levels in intestinal tissue (Yang et al., 2018; Ke et al., 2023; Wang et al., 2012; Wang et al., 2018).

In addition to influence key molecular pathways such as NF-κB, MAPK and PPARγ directly, flavonoids can also affect the Th17/Treg balance by improving the composition of intestinal flora (Kelepouri et al., 2018; Fu et al., 2025). A commensal bacterium, *Bacteroides fragilis*, could inhibit IL-17 production and enhance intestinal Treg cell activity by producing polysaccharide A (PSA) with anti-inflammatory effects (Jin et al., 2012; Round et al., 2011). PSA is an immunomodulatory molecule present in the pod membrane of *B. fragilis*, which mediates the conversion of CD_4_^+^ T cells into Treg cells via toll-like receptor 2 (TLR2) (Round et al., 2011). In addition, PSA is recognized by dendritic cells (DCs) in the intestine and then causes IL-10 production by DC cells, thus promoting Treg production (Chu et al., 2016). Quercetin and luteolin can both decrease the abundance of *B. fragilis*, thereby regulating Th17/Treg balance and cytokine secretion (Yuan et al., 2024; Kelepouri et al., 2018; Fu et al., 2025; Liu et al., 2020; Yang et al., 2025).

Currently, it is still unclear how flavonoids regulate the cytokine profile to make the Th17/Treg axis more balanced in the MAFLD model, such as reducing pro-inflammatory IL-17 and supporting protective IL-22. Future studies should focus on specific flavonoid metabolites and their direct effects on immune cell differentiation and cytokine production within the gut-liver axis to fully characterize these complex interactions. In summary, the synergistic actions of flavonoids with enterocyte and microbial metabolites represent a complex and dynamic regulatory network that can significantly impact hepatic health. By modulating key metabolic pathways and inflammatory responses, these cooperative interactions offer promising therapeutic avenues for the management of MAFLD and its associated complications.

However, while the mechanistic pathways delineated in this section present a compelling framework, the evidence supporting these mechanisms remains largely correlative and derived from imperfect model systems. For instance, the proposed anti-fibrotic effects of quercetin and urolithin A are primarily founded on preclinical models that may not fully recapitulate human disease pathophysiology. A critical, unresolved question is whether the observed microbial shifts (e.g., enrichment of *A. muciniphila* or *Bacteroides* spp.) are related to consequence of improved liver health by flavonoids. This requires causal validation in germ-free or antibiotic depletion animal models to tell whether the absence of gut microbiota abrogates the hepatoprotective effects of flavonoids like naringenin or baicalein. Faecal microbiota transplantation (FMT) studies could also establish whether microbiota from flavonoid-treated donors is sufficient to transfer metabolic benefits. Future research must prioritise these approaches to transcend correlation and establish causality, ensuring that the compelling narrative of flavonoid action via the gut-liver axis is robustly anchored in definitive experimental evidence.

## 4 Clinical evidence and trials

Flavonoids have been explored as potential modulators of the gut-liver axis in the context of metabolic-associated fatty liver disease. Animal studies have shown that flavonoids can modulate the gut microbiota and its metabolites to alleviate MAFLD. Other than preclinical studies in animal models as discussed in previous sections and summarized in Table 3, several clinical studies have documented the efficacy of flavonoids for MALFD management (Table 4).

**TABLE 4:** Clinical trial related to the treatment of MAFLD.

Flavonoid name	Participants	Clinical trial ID	Intervention dose	Clinical endpoints		Key results	References
				Primary outcomes	Secondary outcomes		
Quercetin	41 NAFLD patients (Randomized, Double-Blind, Placebo-Controlled)	ChiCTR2100047904	500 mg/day, oral, 12 weeks	Intrahepatic lipid (IHL) content evaluated by MRI	Liver and renal function, blood lipids and glucose metabolism, body composition and anthropometryetc.	Significantly reduced hepatic fat deposition (MRI-PDFF decreased, p < 0.05)	Li et al. (2024c)
Silymarin	83 MASLD patients (Randomized, Double-Blind, Placebo-Controlled)	ChiCTR2200059043	103.2 mg/day, 6 months	Liver health (including liver stiffness, hepatic steatosis, and liver function)	Metabolic risk factors (including body composition, blood pressure, glucose and lipid profiles, inflammation, and antioxidant capacity)	Reduced liver stiffness (p < 0.01) and correlated with gut microbiota diversity	Jin et al. (2024)
Naringenin	44 NAFLD patients (Randomized, Double-Blind, Placebo-Controlled)	—	200 mg/day, oral, 4 weeks	Improvement of liver steatosis and NAFLD fibrosis score (NFS)	Changes in levels of alanine aminotransferase (ALT), aspartate aminotransferase (AST) and lipid profile	Improved lipid profile, hepatic steatosis severity, and fibrosis probability	Namkhah et al. (2021)

A randomised, double-blind, placebo-controlled crossover clinical trial assessed the impact of quercetin supplementation on intrahepatic lipid content in patients with MAFLD. In this trial, 41 patients were randomised to receive either quercetin (500 mg) or placebo capsules for 12 weeks, followed by a 4-week washout period and subsequent intervention crossover. The primary outcome was intrahepatic lipid content evaluated by magnetic resonance imaging (MRI) estimated proton density fat fraction. Secondary outcomes included liver function measurements and safety assessments. The results showed that quercetin intervention moderately decreased intrahepatic lipid contents from 11.5% ± 6.4%–9.6% ± 5.8%, compared with a minimal decrease of 0.1% ± 2.6% in the placebo group (P = 0.013). Body weight and body mass index (BMI) were also mildly reduced after quercetin intervention (P < 0.05 and adjusted P values of 0.038), while the placebo group experienced much smaller reductions. The reduction in intrahepatic lipid content was positively associated with body weight loss after both interventions. No significant differences were found in other secondary and safety outcomes, and no adverse events were associated with the study intervention. This trial demonstrated that 12 weeks of quercetin treatment could reduce intrahepatic lipid content in MAFLD patients. However, the trial was limited by its relatively small sample size and crossover design, which may have introduced carryover effects despite the washout period. Further trials with larger cohorts and longer intervention durations are needed to confirm these clinical findings and to explore the long-term safety and efficacy of quercetin in MAFLD management (Li N. et al., 2024).

Another randomised, double-blind, placebo-controlled trial registered at the Chinese Clinical Trial Registry (ChiCTR2200059043) investigated the potential efficacy of silymarin in improving MAFLD indicators and the underlying mechanisms related to gut microbiota. In this 24-week trial, 83 patients with MAFLD were randomised to either placebo (n = 41) or silymarin (103.2 mg/d, n = 42). Liver stiffness and hepatic steatosis were assessed using FibroScan at 0, 12, and 24 weeks, while blood samples were collected for biochemical detection and faecal samples were gathered at 0 and 24 weeks for 16S rRNA sequencing. The results showed that silymarin supplementation significantly reduced liver stiffness (LSM, −0.21 ± 0.17 vs. 0.41 ± 0.17, P = 0.015) and serum levels of γ-glutamyl transpeptidase (GGT, −8.21 ± 3.01 vs. 1.23 ± 3.16, P = 0.042), but had no significant effect on other biochemical indicators, physical measurements or fibrosis indices (AST to Platelet Ratio Index and Fibrosis-4 Index). Gut microbiota analysis revealed increased species diversity and enrichment of *Oscillospiraceae* in the silymarin group. These clinical findings suggest that silymarin supplementation could improve liver stiffness in MAFLD patients, possibly by modulating gut microbiota. The trial was limited by its relatively small sample size and the lack of long-term follow-up to assess the sustainability of the observed effects. Further trials are needed to confirm these results and to explore the optimal dosing and duration of silymarin treatment in MAFLD management Meanwhile, the specific mechanism linking gut microbiota changes to liver stiffness was not directly elucidated. Therefore, the findings may have limited generalizability, and future research should focus on confirming these results in larger, more diverse cohorts and exploring the causality of the proposed mechanism (Jin et al., 2024).

Also eligible for randomised, double-blind, placebo-controlled a clinical trial of naringenin included 44 eligible overweight/obese patients with MAFLD. This study assessed the effect of naringenin supplementation on lipid profile, transaminase levels, severity of steatosis and probability of fibrosis. Participants were randomised to receive naringenin capsules (100 mg) or identical placebo capsules twice daily for 4 weeks. The primary outcomes were improvement of liver steatosis and MAFLD fibrosis score (NFS), while secondary outcomes included changes in ALT, AST and lipid profile. The results showed that naringenin consumption significantly reduced the percentages of MAFLD grades (P < 0.001), as well as serum levels of triglyceride (TG) (P < 0.001), total cholesterol (TC) (P = 0.01), and low-density lipoprotein (LDL) (P = 0.02), and increased serum levels of high-density lipoprotein (HDL) (P = 0.02) compared with the control group. However, no significant changes were observed in AST, ALT and NFS. The trial concluded that daily intake of 200 mg of naringenin for 4 weeks had beneficial effects on lipid profile and MAFLD grades as an indicator for the severity of hepatic steatosis (Namkhah et al., 2021).

The aforementioned clinical trials, while providing foundational evidence for the therapeutic potential of flavonoids in MAFLD, are subject to several limitations that warrant careful consideration. A primary and common limitation is the relatively small sample size in all trials (n = 41, n = 83, and n = 44, respectively), with insufficient statistical power to detect smaller, yet clinically meaningful effects, thereby increasing the risk of Type II errors (false negatives), such as failing to identify significant changes in fibrosis scores or other metabolic parameters. Furthermore, the short intervention durations (4, 12, and 24 weeks) are a significant constraint, as meaningful improvements in hepatic fibrosis or metabolic outcomes needs long-term observation, and the reversal of fibrosis typically requires extended periods beyond these timeframes, limiting the ability to capture true therapeutic effects. For instance, the lack of significant change in transaminases and fibrosis scores in the naringenin trial is likely a reflection of its extremely short 4-week duration rather than a true absence of effect, as these markers typically require a longer period to respond to interventions.

Additionally, the reliance on non-invasive surrogate endpoints (e.g., MRI-PDFF for steatosis and FibroScan for stiffness) instead of liver biopsy introduces uncertainty in accurately evaluating the severity of MAFLD and the full extent of histological improvement, as these imaging and biochemical markers may not fully correlate with pathological changes. The crossover design of the quercetin trial, despite its washout period, introduces the potential for carryover effects, where the influence of the initial treatment may persist and confound the results of the subsequent treatment period, a bias that can only be definitively ruled out with a longer washout period or a parallel-group design. The silymarin trial did not establish a direct causal link between the observed changes in *Oscillospiraceae* enrichment and the reduction in liver stiffness. The use of 16S rRNA sequencing also provides only a taxonomic snapshot of the microbiota, lacking the functional insights that could be provided by shotgun metagenomics or metabolomics to track specific flavonoid-derived metabolites. Moreover, these trials did not account for individual variability in flavonoid bioavailability, which is profoundly influenced by gut microbiota composition and genetic background (e.g., polymorphisms in drug-metabolizing enzymes or bile acid receptors); the absence of patient stratification based on enterotypes or genetic markers may obscure subgroup effects and contribute to inconsistent outcomes across studies.

These methodological constraints significantly limit the generalizability of the findings to the broader, heterogeneous MAFLD population, as the specific patient characteristics and baseline disease severity are likely to influence treatment response. Future research must, therefore, be guided by more robust methodologies. This includes large-scale, multi-center, parallel-group randomized controlled trials with intervention periods of at least 6 months to 1 year to properly evaluate sustained efficacy and long-term safety. The inclusion of more definitive clinical endpoints, such as a histological response (via liver biopsy) or significant and sustained changes in liver stiffness (FibroScan) and metabolic markers, will be essential. Furthermore, future studies should incorporate a multi-omics approach to thoroughly investigate the mechanistic link between flavonoids, gut microbiota, and hepatic pathology, moving beyond basic sequencing to measure microbial metabolites and host-derived signalling molecules in the gut-liver axis. Establishing optimal dosing regimens through dose-ranging studies and exploring the therapeutic efficacy in specific patient subgroups (e.g., by genetic polymorphisms or gut enterotypes) will be critical for translating these promising preclinical and preliminary clinical findings into personalized, effective clinical practice.

## 5 Beyond monotherapy: nanotechnology and probiotic Co-administration

### 5.1 Synbiotics: flavonoid-probiotic combinations

As discussed previously, the gut microbiota plays a crucial role in mediating the physiological effects of flavonoids via the gut-liver axis. Probiotics, defined as live microorganisms that confer health benefits when consumed in adequate amounts, can interact synergistically with flavonoids, which are potential prebiotics, to form synbiotics. The combination of dietary flavonoids with specific probiotic strains has emerged as an innovative approach to address gut-liver axis dysregulation in the context of MAFLD (Jazvinšćak et al., 2023). Probiotic enzymatic activity activates flavonoid precursors into bioactive metabolites, while flavonoids selectively modulate the gut microbiota and microbial composition, creating a self-amplifying loop that enhances MAFLD management. This positive feedback loop targets multiple pathological mechanisms and exerts beneficial effects on oxidative stress, inflammation, and the gut microbiome (Zhu et al., 2022). In this section, we review several studies investigating the combined effects of flavonoids and probiotics on MAFLD, elucidating their therapeutic potential.

#### 5.1.1 Quercetin and *Akkermansia muciniphila*

*Akkermansia muciniphila* is a prominent gut bacterium that has shown potential in improving metabolic diseases, including obesity and MAFLD. In a landmark study involving HFD-induced obese mice, researchers demonstrated that the synbiotic combination of *A. muciniphila* with quercetin was superior in reducing hepatic steatosis and insulin resistance compared to either intervention alone. This combination restored intestinal barrier integrity, as evidenced by upregulation of tight junction proteins, such as Claudin-1 and Occludin, while decreasing LPS translocation (Le Barz et al., 2019). Moreover, the combination reshaped the microbiota by enriching *Roseburia* and *Faecalibacterium*, bacteria that produce butyrate, a SCFA with anti-inflammatory properties. Notably, the quercetin-*A. muciniphila* synergy also modulated BA metabolism with enhanced FXR signalling, which suppressed hepatic lipogenesis by downregulating SREBP-1c and activated mitochondrial β-oxidation by upregulating PGC-1α. Co-administration of quercetin (50 mg/kg/day) with *A. muciniphila* (1 × 10^9^ CFU/day) resulted in a 38% greater reduction in hepatic TG content compared to either treatment alone. This highlights the potential of flavonoid-probiotic combinations to modulate multiple metabolic pathways and improve liver health in MAFLD (Juárez-Fernández et al., 2021).

#### 5.1.2 Grapeseed flour and *Lactobacillus acidophilus*

Grapeseed flour (GSF) is rich in flavonoids, particularly proanthocyanidins, which possess potent antioxidant and anti-inflammatory properties (Cho et al., 2018). In a 24-week clinical trial, a synbiotic combination of GSF and kefir-derived probiotics *L. acidophilus LA-5* was showed to reduced liver fat content, as measured by the controlled attenuation parameter, by 22% compared to the use of probiotics alone. Additionally, this combination downregulated hepatic SREBP-1c (62%) and ACC (55%) expression through AMPK phosphorylation, as validated by CRISPR-Cas9 knockout models. The synbiotic treatment also reversed HFD-induced gut barrier dysfunction, which is crucial for preventing endotoxemia and inflammation in MAFLD. Moreover, *Lactobacillus acidophilus* enhanced the absorption of procyanidins by degrading mucus-bound glycoproteins in the intestine, thereby improving their bioavailability. These clinical findings suggest that combining flavonoid-rich GSF with probiotics can significantly ameliorate hepatic steatosis and related metabolic disturbances (Kwon et al., 2019; Seo et al., 2020).

#### 5.1.3 Green tea EGCG and *Lactobacillus fermentum*

Epigallocatechin gallate (EGCG), a flavonoid extracted from green tea, has been extensively studied for its antioxidant and anti-inflammatory properties, both of which are essential for the management of MAFLD (Carrasco-Pozo et al., 2019; Talib et al., 2024). In a study involving aged C57BL/6 mice with diet-induced MAFLD, a synbiotic combination of EGCG and the probiotic *L. fermentum* was tested for its protective effects against oxidative stress and inflammation (Sharma et al., 2019). The synbiotic was found to increase hepatic glutathione peroxidase activity by 40%, decrease malondialdehyde (a marker of lipid peroxidation) level by 35%, and suppress CD8^+^ T-cell hepatic infiltration, which is indicative of liver inflammation. Transcriptome analysis revealed upregulation of antioxidant genes (HO-1, NQO1) and downregulation of Th17-related cytokines, suggesting that the synbiotic combination modulated both oxidative stress and immune responses (Ting et al., 2022). The EGCG component inhibited JNK phosphorylation, a key pathway in oxidative stress, while *Lactobacillus fermentum* promoted T-regulatory cell proliferation, further supporting the anti-inflammatory effects (Dey et al., 2020). The strong upregulation of hepatic Nrf2 expression exclusively in the synbiotic-fed animals provides additional evidence of the robust antioxidant defence induced by EGCG-*L. fermentum* combination (Sharma et al., 2019). These results highlight the potential of flavonoid-probiotic synbiotics as a therapeutic strategy for MAFLD, particularly in populations prone to oxidative stress, such as the elderly (Sharma et al., 2019; Huang et al., 2020).

#### 5.1.4 Flavonoid-probiotic synbiotics: challenges and future

In conclusion, flavonoid-probiotic combinations represent a promising avenue for the treatment of MAFLD. The synergistic effects of these bioactive compounds, targeting multiple pathways such as gut microbiota modulation, bile acid metabolism, and hepatic lipid metabolism, provide a multifaceted cutting-edge strategy to managing this increasingly prevalent liver disease (Axling et al., 2012; Xiong et al., 2023; Thilakarathna and Rupasinghe, 2024; Peng et al., 2020). However, existing studies highlight several challenges, most notably the critical issue of strain-specificity where effects observed with particular bacterial strains (e.g., *Lactobacillus* or *B. spp*.) cannot be extrapolated to the entire species, yet many studies fail to adequately characterize or report the specific strains utilised, rendering results difficult to interpret and replicate. Other challenges include the need for optimal strain selection, biosafety evaluation, and the impact of patient variability, including genetic predisposition, individual microbiome composition, and dietary habits, on therapeutic outcomes (DiStefano, 2023; Oh et al., 2023; Wang M. et al., 2022; Ribeiro et al., 2018). Furthermore, there is a conspicuous lack of standardisation in formulations and dosages across studies, with highly variable flavonoid-to-probiotic ratios, delivery formats, and intervention regimens creating significant obstacles for comparing outcomes and establishing reproducible therapeutic protocols. Future research must focus on strain-specific screening, pharmacokinetic modelling, and long-term safety assessments to ensure the clinical translation of these promising preclinical findings (Palencia-Argel et al., 2024).

Genetic polymorphisms have also been identified as crucial determinants of the therapeutic response to flavonoid-based treatments, such as silibinin. For instance, Lrp6(+/−) mice exhibited less severe liver injury in response to MCD, but a reduced treatment response to silibinin, compared to Lrp6(+/+) mice, suggesting that Lrp6 may serve as a target for silibinin’s therapeutic action (Chen et al., 2021). This highlights the need for personalized treatment approaches based on genetic variations, as individual susceptibility to MAFLD may be modulated by specific genetic factors, including Lrp6 polymorphisms (Chen et al., 2021). Addressing these issues will be critical in ensuring that flavonoid-probiotic combinations can be successfully used as personalized therapeutic strategies for MAFLD.

Despite the compelling preclinical evidence, the clinical translation of flavonoid-probiotic synbiotics encounters substantial hurdles. A particularly significant barrier is the regulatory gap whereby these combinations are typically classified as dietary supplements or probiotics rather than pharmaceuticals, subjecting them to considerably less stringent requirements for demonstrating efficacy, safety, and quality control compared to medicinal products—this permissive and inconsistent regulatory landscape contributes to variable product quality and weakened clinical evidence. A primary unmet need lies in establishing standardized formulations and dosages, given the immense diversity of flavonoid compounds and probiotic strains, which contributes to highly variable synergistic effects *in vivo* (Palencia-Argel et al., 2024). The intricate interplay among distinct flavonoid types, specific probiotic strains, individual host microbiomes, and dietary factors necessitates extensive and rigorous clinical trials to ascertain consistent efficacy and safety in MAFLD patients. Furthermore, the evolving regulatory requirement for synbiotic products presents additional challenges for their widespread clinical adoption and therapeutic standardization. Therefore, while these combinations hold considerable promise, achieving clinical readiness mandates overcoming these multifaceted translational gaps through meticulously designed, large-scale clinical studies and the establishment of clearer regulatory frameworks.

### 5.2 Nanotechnology for targeted delivery

Flavonoids exhibit broad-spectrum biological and pharmacological properties, but many of their key constituents is limited by physicochemical constraints such as poor dispersibility, and instability, as well as extensive gastrointestinal degradation, liver first-pass metabolism, and restricted membrane transport, all collectively contributing to reduced oral bioavailability (Bhia et al., 2021; Zhang et al., 2022). Nanotechnology, including flavonoid-loaded nanoparticles like chitosan, nanoliposomes, and solid lipid nanoparticles, has been developed to enhance the bioavailability, stability, solubility, and delivery of flavonoids such as EGCG (Hu L. et al., 2025; Prananda et al., 2025; Shi et al., 2018).

Chitosan nanoparticles, in particular, have been widely used to encapsulate flavonoids due to their biocompatibility, biodegradability, and mucoadhesive properties (Prananda et al., 2025; Seyam et al., 2020). These nanoparticles can be designed to provide controlled and sustained release of encapsulated flavonoids, ensuring a gradual and prolonged delivery to target cells or tissues (Stevens Barron et al., 2023). For example, one study focused on the effect of chitosan-modified, silymarin-loaded lipid-polymer hybrid nanoparticles (CS-LPNs) in enhancing the oral bioavailability of silymarin and improving its lipid-lowering efficacy for NAFLD treatment. The results showed that the relative bioavailability of CS-LPNs was 14.38 times higher than that of silymarin suspension, and it enhanced the uptake of the nanocarriers by fat-emulsion-treated HepG2 and Caco-2 cells. Meanwhile, the study confirmed that CS-LPNs inhibited lipid accumulation in the mouse liver and enhanced the therapeutic efficacy of silymarin in a transgenic mouse model of NAFLD. These clinical findings suggest that the improved uptake of CS-LPNs could be achieved *in vivo*, potentially increasing the oral bioavailability of silymarin (Talib et al., 2024; Liang et al., 2018). Also, in the field of lipid reduction, a recent study explored a novel strategy for obesity treatment by using hydroxy-α-sanshool-loaded adipose-targeted mesoporous silica nanoparticles (MSNs) to specifically induce the browning of white adipose tissue (WAT). This research demonstrated that the nanocarriers activate the transient receptor potential vanilloid 1 (TRPV1) channel, providing a potential therapeutic approach for MAFLD patients with hepatic lipid accumulation (Zhang Q. et al., 2025).

Nanoliposomes have also been used to encapsulate flavonoids, such as EGCG, to improve their bioavailability and stability (Talib et al., 2024; Hu L. et al., 2025). These nanoparticles can be designed to provide targeted delivery of flavonoids to specific tissues, such as the myocardium or vascular endothelium, via endocytotic mechanisms (Prananda et al., 2025). Additionally, nanoliposomes have been shown to improve the therapeutic efficacy of flavonoids by enhancing their absorption and distribution in the body (Talib et al., 2024; Hu L. et al., 2025; Kasem et al., 2025).

While nanotechnology presents significant promise for overcoming the inherent limitations of flavonoid delivery, its clinical translation is fraught with several critical caveats and unmet needs. The vast majority of existing research remains confined to pre-clinical stages, with the long-term biosafety profile of these nanocarriers (including CS-LPNs and MSNs) representing a formidable “area of unknown” in clinical translation. Comprehensive assessments of their potential for long-term accumulation, immunogenicity, and unanticipated organ toxicity following chronic administration are notably lacking, necessitating extensive *in vivo* investigations to preclude unanticipated adverse effects, particularly with chronic administration (Bhia et al., 2021; Zhang et al., 2022; Hu L. et al., 2025). Furthermore, the challenges pertaining to reproducible synthesis, scalable manufacturing, and cost-effectiveness of nano formulations must be rigorously addressed for viable clinical applications (Shi et al., 2018). Most current protocols remain at the proof-of-concept stage, with little consideration given to Good Manufacturing Practice (GMP) compliance or industrial scalability, whilst stability issues during storage and transport present additional hurdles for real-world implementation. Meanwhile, the formidable biological barriers and complex *in vivo* interactions imply that targeted delivery, though conceptually appealing, is often less efficient than *in vitro* results. This disparity underscores the need for more sophisticated targeting strategies and meticulous preclinical validation.

## 6 Future perspective

The growing body of evidence highlights flavonoids as potential therapeutic agents for MAFLD, but significant challenges remain in translating these findings into effective clinical practice (Qian et al., 2024; Nie et al., 2025). A critical hurdle is addressing the significant inter-individual variability in response to flavonoid interventions. The complex interplay between flavonoids and the gut microbiota, shaped by host genetics, dictates their metabolism and bioavailability. Future research must move beyond a one-size-fits-all approach to investigate these genetic and microbial factors to develop more personalized strategies.

Another important caveat in developing flavonoid-based MAFLD drugs is that many natural flavonoids fall into the category of Pan-Assay Interference Compounds (PAINS) (Baell, 2016). These compounds often contain problematic motifs, such as redox-active catechols or aggregatory structures, which require cautious interpretation of data from high-throughput *in vitro* or cell-based assays. Therefore, structure-activity relationship (SAR) studies should aim to optimize flavonoids toward improved drug-like properties by reducing polyphenolic character while maintaining efficacy. Ultimately, demonstrating target engagement in physiologically relevant animal models is critical to distinguish true pharmacology from assay artifacts. Only rigorous, evidence-based approaches ensure that promising flavonoids advance as *bona fide* therapeutic candidates rather than as PAINS-misleading hits.

The translation of flavonoid-based therapies from bench to bedside for MAFLD management is primarily hampered by two interconnected barriers: the scarcity of robust long-term clinical evidence and the inherent challenges of bioavailability and consistent efficacy. The considerable inter-individual variation in flavonoid metabolism, heavily influenced by unique host genetics and gut microbial composition, necessitates a fundamental shift away from universal dosing regimens towards personalized, biomarker-guided approaches. Future clinical trials must therefore be larger, longer, and integratively designed to incorporate multi-omics analyses (metagenomics, metabolomics, genomics), enabling patient stratification and the identification of predictive biomarkers for treatment response.

To overcome the bioavailability barrier, innovative delivery systems such as nanotechnology and synbiotic formulations present promising avenues. However, their clinical translation mandates a critical focus on overcoming hurdles related to long-term safety, reproducible large-scale manufacturing, and the establishment of standardized regulatory frameworks. Ultimately, the future of flavonoid therapy lies in leveraging advanced delivery technologies and precision nutrition principles to transform the gut-liver axis paradigm into tangible, effective, and personalized clinical strategies for MAFLD.

To effectively translate these findings into clinical practice, a shift in perspective is required for both healthcare providers and researchers. Clinicians must move toward a personalized approach, using advanced diagnostic tools to screen for genetic predispositions and gut microbiome composition to identify which patients will benefit most. Researchers, in turn, should prioritize developing standardized, reproducible methods for assessing flavonoid bioavailability and metabolism in humans through well-powered, long-term randomized controlled trials. These efforts should focus on understanding dose-response relationships and developing safe, effective delivery systems. By overcoming these barriers, flavonoids could provide a novel, multi-targeted strategy to manage the complex pathophysiology of MAFLD/MASH, potentially revolutionizing treatment options.

## Glossary

ACACA: Acetyl-CoA Carboxylase Alpha
ACC: Acetyl-CoA Carboxylase
ALK5: TGF-β type I receptor
AREs: antioxidant response elements
BA: bile acid
BMI: body mass index
BSH: bile salt hydrolases
ChREBP: carbohydrate-responsive element-binding protein
CPT1: Carnitine Palmitoyltransferase 1
CS-LPNs: chitosan-modified, silymarin-loaded lipid-polymer hybrid nanoparticles
CYP2E1: cytochrome P450 2E1
CYP7A1: cytochrome P450 7A1
DNL: *de novo* lipogenesis
ECM: extracellular matrix
EGCG: Epigallocatechin gallate
FA: fatty acid
FAO: fatty acid oxidation
FASN: Fatty Acid Synthase
FGF19: fibroblast growth factor 19
FGFR4: FGF19 receptor
FLR: flavone reductase
GCLC: glutamate-cysteine ligase catalytic subunit
GGT: γ-glutamyl transpeptidase
GPCRs: G-protein coupled receptors
GSH: glutathione
GSF: Grapeseed flour
HCC: hepatocellular carcinoma
HDACs: histone deacetylases
HDL: high-density lipoprotein
HFD: high-fat diet-fed
HO-1: Heme Oxygenase-1
HSCs: Hepatic Stellate Cells
IECs: Intestinal Epithelial Cells
IL-6: interleukin-6
JNK: c-Jun N-terminal kinase
KCs: Kupffer cells
LDL: low-density lipoprotein
LPS: lipopolysaccharides
LRP5/6: low-density lipoprotein receptor-related protein 5/6
MAFLD: Metabolic dysfunction-associated fatty liver disease
MAPK: mitogen-activated protein kinase
MASH: Metabolic dysfunction-associated steatohepatitis
MDA: malondialdehyde
MRI: magnetic resonance imaging
NFS: MAFLD fibrosis score
NHANES: National Health and Nutrition Examination Survey
NQO1: NAD(P)H quinone oxidoreductase 1
Nrf2: nuclear factor erythroid 2-related factor 2
PMFs: Polymethoxyflavones
PPARs: peroxisome proliferator-activated receptors
PPARα: Peroxisome Proliferator-Activated Receptor alpha
PSA: polysaccharide A
ROS: reactive oxygen species
SCFAs: Short-chain fatty acids
SFB: Segmentous filamentous bacteria
SREBP-1c: Sterol Regulatory Element-Binding Protein 1c
TC: total cholesterol
TG: triglyceride
TGF-β: Transforming growth factor-beta
TLR2: Toll-like receptor 2
TLR4: Toll-like receptor 4
Treg: T regulatory
